# Suppression of Hepatocyte Ferroptosis via USP19‐Mediated Deubiquitination of SLC7A11 in Ischemia‐Free Liver Transplantation

**DOI:** 10.1002/advs.202406200

**Published:** 2024-11-22

**Authors:** Jinghong Xu, Shirui Chen, Di Liu, Qi Zhang, Tao Luo, Jiaxing Zhu, Liang Zhou, Yuan Lin, Hongyu Pan, Yichao Chen, Qiang Zhao, Tielong Wang, Schlegel Andrea, Björn Nashan, Tullius G. Stefan, Changjie Cai, Jun Cui, Xiaoshun He, Zhiyong Guo

**Affiliations:** ^1^ Organ Transplant Center The First Affiliated Hospital Sun Yat‐sen University Guangzhou Guangdong 510080 China; ^2^ Department of Anesthesiology The First Affiliated Hospital Sun Yat‐sen University Guangzhou Guangdong 510080 China; ^3^ Guangdong Provincial Key Laboratory of Organ Medicine Guangzhou Guangdong 510080 China; ^4^ Guangdong Provincial International Cooperation Base of Science and Technology (Organ Transplantation) Guangzhou Guangdong 510080 China; ^5^ Department of Thyroid and Breast Surgery The Second Affiliated Hospital of Anhui Medical University Hefei Anhui 230601 China; ^6^ Zhongshan School of Medicine Sun Yat‐sen University Guangzhou Guangdong 510080 China; ^7^ School of Life Sciences Sun Yat‐sen University Guangzhou Guangdong 510080 China; ^8^ Department of Pathology The First Affiliated Hospital Sun Yat‐sen University Guangzhou Guangdong 510080 China; ^9^ Transplantation Center Digestive Disease and Surgery Institute and Department of Immunology Lerner Research Institute, Cleveland Clinic Cleveland Ohio 44113 USA; ^10^ Organ Transplant Center The First Affiliated Hospital of the University of Science and Technology of China Hefei Anhui 230001 China; ^11^ Division of Transplant Surgery Brigham and Women's Hospital Harvard Medical School Boston MA 02115 USA; ^12^ Department of Critical Care The First Affiliated Hospital Sun Yat‐sen University Guangzhou Guangdong 510080 China; ^13^ NHC Key Laboratory of Assisted Circulation Sun Yat‐sen University Guangzhou Guangdong 510080 China

**Keywords:** ferroptosis, ischemia reperfusion injury, liver transplantation, ubiquitin specific protease 19

## Abstract

Ischemia‐free liver transplantation (IFLT) is developed as a novel clinical approach to avoid ischemia‐reperfusion injury (IRI). This study aims to identify the most distinguished programmed cell death pathway in grafts undergoing IFLT versus conventional liver transplantation (CLT) and to explore the underlying mechanism. Ferroptosis is the most distinct programmed cell death form between IFLT and CLT grafts. Among various cell death inhibitors, the ferroptosis inhibitor (Ferrostain‐1) is the most effective one to prevent hepatocytes from damage induced by oxygen deprivation/reoxygenation (OGD/R). Hepatocyte ferroptosis is significantly alleviated in IFLT versus CLT grafts in both human beings and pigs. Ubiquitination enzyme screening identifies augmented amounts of ubiquitin‐specific protease 19 (USP19) in IFLT versus CLT grafts. The upregulation of USP19 in the grafts is correlated with reduced pathological Suzuki's score, lower post‐transplant peak liver enzyme level, and less early allograft dysfunction in liver transplant recipients. USP19 overexpression mitigates post‐transplant liver injury in mice. Mechanistically, USP19 inhibits the degradation of solute carrier family 7 member 11 (SLC7A11) by removing its K63‐linked ubiquitin chains. Notably, USP19 overexpression reduces ferroptosis and IRI in a SLC7A11‐dependent manner in mice. Collectively, USP19‐mediated suppression of hepatocyte ferroptosis via deubiquitinating SLC7A11 is a key mechanism by which IFLT abrogates graft IRI.

## Graphical abstract

Compared with conventional liver transplantation, the expression of USP19 increases in ischemia‐free liver transplantation (IFLT) grafts. Overexpression of USP19 alleviates hepatocyte ferroptosis and graft ischemia‐reperfusion injury (IRI). USP19 protects SLC7A11 from autophagic degradation by removing K63‐linked ubiquitination, promoting ROS scavenging and reducing ferroptosis. Collectively, IFLT mitigates IRI by inhibiting ferroptosis and USP19 is a potential therapeutic target of ferroptosis.

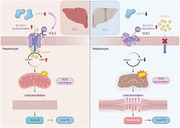

## 1 Introduction

Liver transplantation is the standard therapy for end‐stage liver disease. During conventional liver transplantation (CLT), the donor liver is procured after a cold flush, preserved, and transported usually in a cold (4 °C) preservation solution, and then implanted under hypothermic and hypoxic conditions. The subsequent reperfusion after implantation inevitably introduces graft ischemia‐reperfusion injury (IRI), resulting in a vast range of complications including post‐reperfusion syndrome, early allograft dysfunction, primary non‐function, and ischemia‐type biliary lesions.^[^ ^1^, ^2^ ^]^

IRI is a two‐stage phenomenon associated with the reduction of blood flow to an organ, resulting in hypoxia and cell damage, which is then exacerbated upon restoration of oxygen delivery.^[^ ^3^ ^]^ Although numerous interventions can ameliorate IRI in mice, these strategies are merely translated into clinical practice. Recently, various types of machine perfusion have been used to preserve donor livers.^[^ ^4^, ^5^ ^]^ The feasibility and efficacy of machine perfusion in the reduction of graft IRI have been shown in clinical trials.^[^ ^6^, ^7^ ^]^ The consequences of injury may be reduced with those approaches. However, graft IRI is still present as machine perfusion is only used during the preservation stage and the livers suffer ischemia prior to machine perfusion and during implantation.^[^ ^8^, ^9^ ^]^ Our group has proposed a new concept of ischemia‐free liver transplantation (IFLT) during which the donor liver is procured, preserved, and implanted under continuous normothermic oxygenated conditions.^[^ ^10^, ^11^ ^]^ We have shown not only the feasibility of IFLT but also the advantages of this approach by transplanting a donor liver with over 85% macrovesicular steatosis.^[^ ^11^ ^]^ Our recent randomized trial has demonstrated that IFLT significantly reduces IRI‐related complications.^[^ ^12^ ^]^ Of particular relevance, histological, transcriptomic, and metabolomic analysis of liver biopsies documents almost absence of graft IRI in IFLT.^[^ ^13^ ^]^ IFLT provides therefore not only an entirely novel approach to avoid IRI but also a unique model to investigate the mechanisms of graft IRI during liver transplantation.

Activation of various cell death pathways is one of the key mechanisms of hepatic IRI. Ferroptosis is morphologically, genetically, and biochemically distinct from other recognized cell death pathways.^[^ ^14^ ^]^ Morphologically, ferroptosis cells show typical necrosis‐like changes.^[^ ^15^ ^]^ Biochemically, ferroptosis is characterized by the production of lethal levels of iron‐dependent lipid peroxidation.^[^ ^16^ ^]^ The solute carrier family 7 member 11 (SLC7A11)‐glutathione (GSH)‐glutathione peroxidase 4 (GPX4) axis is considered critical in the prevention of ferroptosis.^[^ ^17^, ^18^, ^19^ ^]^ Ubiquitination represents a reversible form of protein post‐translational modification (PTM). Dynamic changes between ubiquitination and deubiquitination can regulate protein abundance, localization, and activity, thereby affecting cell survival and death.^[^ ^20^ ^]^

In the current study, we found that ferroptosis is the most distinct programmed cell death form between IFLT and CLT grafts. Ferroptosis is substantially reduced in IFLT versus CLT grafts. Mechanically, ubiquitin‐specific protease 19 (USP19) expression is upregulated in IFLT grafts with the ability to suppress ferroptosis by deubiquitinating SLC7A11.

## 2 Results

### 2.1 IFLT Reduces IRI and Suppresses Hepatocyte Ferroptosis

**Figure 1:**
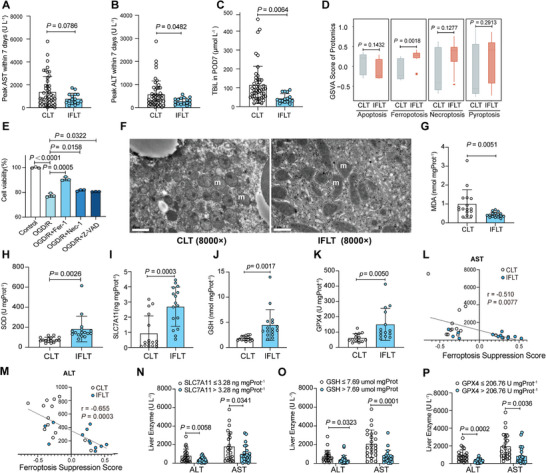
IFLT reduces IRI and suppresses ferroptosis in clinical practice. A–C) IFLT reduces IRI. The peak serum AST (A) and ALT (B) within 7 days and TBIL (C) on day 7 post‐liver transplantation were compared between IFLT and CLT groups (IFLT, *n* = 16; CLT, *n* = 48). D) The differences in GSVA scores between the four programmed cell death pathways are shown. E) Viability of mouse hepatocytes following OGD/R and treatment with inhibitors of apoptosis (Z‐VAD), ferroptosis (Fer‐1), and necrosis (Nec‐1). Z‐VAD (10 µM), Fer‐1 (5 µM), or Nec‐1 (10 µm) were administered to the hepatocyte culture medium prior to subjecting the hepatocytes to 4 h of oxygen deprivation followed by 4 h of reoxygenation. Finally, cell viability was assessed. F) Ultrastructural analysis of liver biopsy from CLT and IFLT. m, mitochondrion. scale bar, 1 µm; × 8000 magnification. G,H), MDA (G), and SOD (H) were compared between CLT and IFLT liver biopsies (IFLT, *n* = 16; CLT, *n* = 48). I–K) The ferroptosis negative regulation axis of SLC7A11 (I)‐GSH (J)‐ GPX4 (K) were measured in both IFLT and CLT liver tissues (IFLT, *n* = 16; CLT, *n* = 48). L,M) The correlation analysis between ferroptosis suppression score and the peak serum AST (L) and ALT (M) within 7 days after liver transplantation in IFLT and CLT groups (IFLT, *n* = 16; CLT, *n* = 48). N–P) The peak serum AST and ALT were compared between the SLC7A11 (N), GSH (O), and GPX4 (P) high‐ and low‐expression groups (*n* = 48). The error bars represent the standard deviation of the indicated dataset. P values in (A–E), (G–K), and (N–P) were analyzed by unpaired two‐tailed Student's t‐test (Prism; GraphPad), and (L & M) were analyzed by linear regression algorithm.

Our previous trial has shown both feasibility and safety of IFLT.^[^ ^12^ ^]^ In the current study, we retrospectively included recipients who underwent IFLT or CLT at our hospital from July 2021 to May 2022 (Figure S1 and Table S1, Supporting Information). The levels of postoperative serum liver enzymes and bilirubin were compared between included 16 IFLT and 48 CLT cases. We found that the peak levels of aspartate aminotransferase (AST, 778.75 ± 472.23 U L^−1^ vs 1392.63 ± 1339.61 U L^−1^, *P* = 0.0786) and alanine aminotransferase (ALT, 281.69 ± 144.11 U L^−1^ vs 578.29 ± 579.96 U L^−1^, *P* = 0.0482) within 7 days post‐transplantation were reduced by 44.1% and 51.3% in IFLT compared to CLT recipients (**Figure** **1**A,B). Additionally, total bilirubin levels (44.49 ± 24.50 µmol L^−1^ vs 115.35 ± 99.00 µmol L^−1^, *P* = 0.0064) on day 7 post‐transplantation declined by 61.4% in IFLT versus CLT recipients (Figure 1C).

Considering that hepatocyte death is one of the direct victims and drivers of IRI,^[^ ^16^, ^21^ ^]^ we calculated the Gene Set Variation Analysis (GSVA) scores of programmed cell death patterns by enriching peptide fragments using our previously published proteomics database.^[^ ^13^ ^]^ Among the four programmed cell death types (apoptosis, necroptosis, pyroptosis, and ferroptosis), ferroptosis was found to be the most differently displayed pathway between IFLT and CLT grafts (Figure 1D). The list of genes directly related to necroptosis, apoptosis, pyroptosis, and ferroptosis are summarized in Table S2 (Supporting Information). We then co‐treated primary mouse hepatocytes with death inhibitors and found that the inhibitors of apoptosis (benzyloxycarbonyl‐Vad‐Ala‐Asp‐fluoromethyl ketone, Z‐VAD), ferroptosis (Ferrostain‐1, Fer‐1), and necrosis (Necrostain‐1, Nec‐1) all failed to completely block oxygen deprivation/reoxygenation (OGD/R)‐induced cell death of hepatocytes. Nevertheless, the ferroptosis inhibitor exhibited the most pronounced effect in alleviating cell damage (Figure 1E; Figure S2, Supporting Information), suggesting that ferroptosis might be the dominant form of cell deaths during OGD/R of primary hepatocytes.

Next, we compared the level of ferroptosis in donor liver biopsy samples. As shown in the transmission electron microscope (TEM) images (Figure 1F), mitochondria were shrinking, with higher density and reduced cristae in CLT compared to IFLT grafts. Moreover, levels of malondialdehyde (MDA), the product of lipid peroxidation, were reduced in IFLT versus CLT liver grafts (0.43 ± 0.14 nmol mgProt^−1^ vs 1.00 ± 0.74 nmol mgProt^−1^, *P* = 0.0051) (Figure 1G). Notably, superoxide dismutase (SOD), an antioxidant enzyme that can prevent peroxidation‐induced ferroptosis, was significantly elevated in IFLT versus CLT grafts (182.76 ± 127.75 U mgProt^−1^ vs 75.72 ± 24.87 U mgProt^−1^, *P* = 0.0026) (Figure 1H). Furthermore, levels of anti‐ferroptosis proteins SLC7A11 (2.69 ± 1.30 ng mgProt^−1^ vs 0.94 ± 1.14 ng mgProt^−1^, *P* = 0.0003), GSH (4.48 ± 3.06 nmol mgProt^−1^ vs 1.82 ± 0.47 nmol mgProt^−1^, *P* = 0.0017) and GPX4 (150.35 ± 104.58 U mgProt^−1^ vs 61.63 ± 28.14 U mgProt^−1^, *P* = 0.0050) were significantly elevated in IFLT versus CLT grafts (Figure 1I–K), suggesting that the negative regulatory axis of ferroptosis is of importance for the ameliorated consequences of IRI observed in IFLT.

Next, to delineate the effects of ferroptosis in liver IRI, we applied a Ferroptosis Suppression Score based on ferroptosis suppressor genes detected in our transcriptome database by utilizing the GSVA algorithm, thus reflecting the activation of the negative regulatory pathway of ferroptosis.^[^ ^22^ ^]^ We found that the Ferroptosis Suppression Score was significantly elevated in IFLT versus CLT grafts (0.14 ± 0.31 vs −0.25 ± 0.23, *P* = 0.0003). Moreover, the score correlated negatively with the peak AST (r = −0.510, *P* = 0.0077) and ALT (r = −0.655, *P* = 0.0003) levels within the first 7 days post‐transplantation (Figure 1L,M). We further divided the 48 CLT cases into low‐expression and high‐expression groups based on the median intragraft expression levels of SLC7A11 (3.28 ng mgProt^−1^), GSH (7.69 nmol mgProt^−1^), and GPX4 (206.76 U mgProt^−1^). Patients in the high‐expression groups of SLC7A11, GSH, and GPX4 had lower peak AST and ALT levels than those in the low‐expression groups (Figure 1N–P). Collectively, these results suggest that suppression of ferroptosis represents a key mechanism by which IFLT abrogates graft IRI.

**Figure 2:**
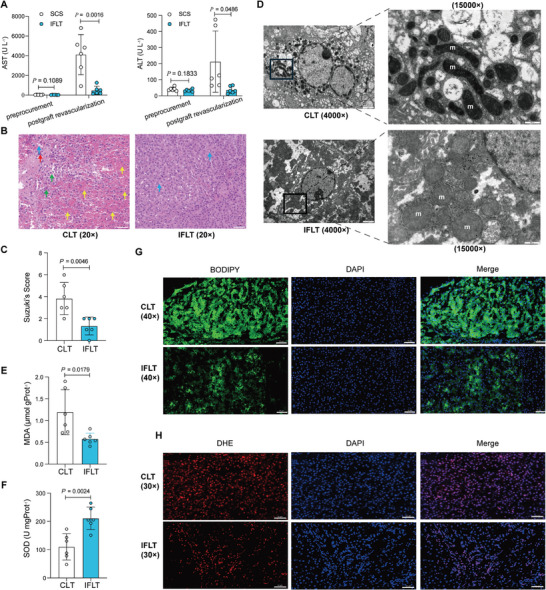
IFLT reduces IRI and suppresses ferroptosis in pigs. A–C), IFLT attenuates IRI. Liver injury was compared between IFLT and CLT groups (*n* = 6 for each group) by AST, ALT levels (A) and pathological manifestations (B & C) at 6 h post‐implantation. Red arrows, infiltrating inflammatory cells; green arrows, swelling of hepatocytes with loose cytoplasm and light staining; blue arrows, balloon degeneration, cytoplasmic vacuolization; yellow arrows, visible hemorrhage within the field of view. scale bars, 50 µm, × 20 magnification. D) The mitochondrial ultrastructure was captured by TEM. m, mitochondrion. left panel: scale bars, 2 µm, × 4000 magnification; right panel: scale bars, 500 nm, × 15 000 magnification. E,F) Levels of lipid peroxidation in livers were measured by MDA and SOD assay. G,H) Representative images of the fluorescence assay of ROS in livers using the fluorescent probe C11‐BODIPY (green) and DHE (red), with nuclei stained with DAPI (blue). Scale bar, 50 µm.

To minimize inter‐individual heterogeneity, we established IFLT and CLT models in pigs. Consistent with the results in clinical practice, 6 h after liver implantation, the levels of AST (483.82 ± 424.01 U L^−1^ vs 4119.75 ± 2033.41 U L^−1^, *P* = 0.0016) and ALT (37.00 ± 22.05 U L^−1^ vs 212.67 ± 190.41 U L^−1^, *P* = 0.0486) were significantly lower in the IFLT group than in the CLT group (**Figure** **2**A). Morphologically, hepatic IRI in pigs was observed using hematoxylin and eosin (H&E) staining and evaluated by Suzuki's classification. The livers in the CLT group showed severe congestion, vacuolation, and hepatocyte necrosis, whereas the livers in the IFLT group showed milder changes (Figure 2B). Moreover, grafts in the IFLT group showed lower Suzuki's scores (1.33 ± 0.82 vs 3.83 ± 1.47, *P* = 0.0046) compared to those in the CLT group (Figure 2C). Ultrathin sections were prepared from donor's livers at 6 h post‐implantation, revealing pronounced mitochondrial shrinkage in the soma of hepatocytes in the CLT group (Figure 2D). The occurrence of ferroptosis was also evaluated by lipid peroxidation manifested by MDA (Figure 2E) and SOD (Figure 2F) content. The MDA concentration in the IFLT group was conspicuously lower than that in the CLT group (0.58 ± 0.13 µmol gProt^−1^ vs 1.19 ± 0.51 µmol gProt^−1^, *P* = 0.0179) (Figure 2E), whereas SOD exhibited a contrary trend (210.79 ± 39.91U mgProt^−1^ vs 110.06 ± 46.59 U mgProt^−1^, *P* = 0.0024) (Figure 2F). Reactive oxygen species (ROS) are generated to induce lipid peroxidation during IRI, representing a critical molecular pathway of hepatic injury.^[^ ^23^, ^24^ ^]^ C11‐BODIPY and dihydroethidium (DHE), serving as a fluorescent probe, were used to characterize intracellular lipid ROS. Staining images obtained using the C11‐BODIPY 581/591 and DHE fluorescence probe showed increased green/red fluorescence in the CLT versus IFLT grafts, indicating greater lipid ROS production in hepatocytes of CLT versus IFLT grafts (Figure 2G,H). Collectively, these results indicate that IFLT, a novel transplant technique, could attenuate ferroptosis during liver graft IRI.

### 2.2 Deubiquitinase USP19 Suppresses Ferroptosis of Hepatocytes

**Figure 3:**
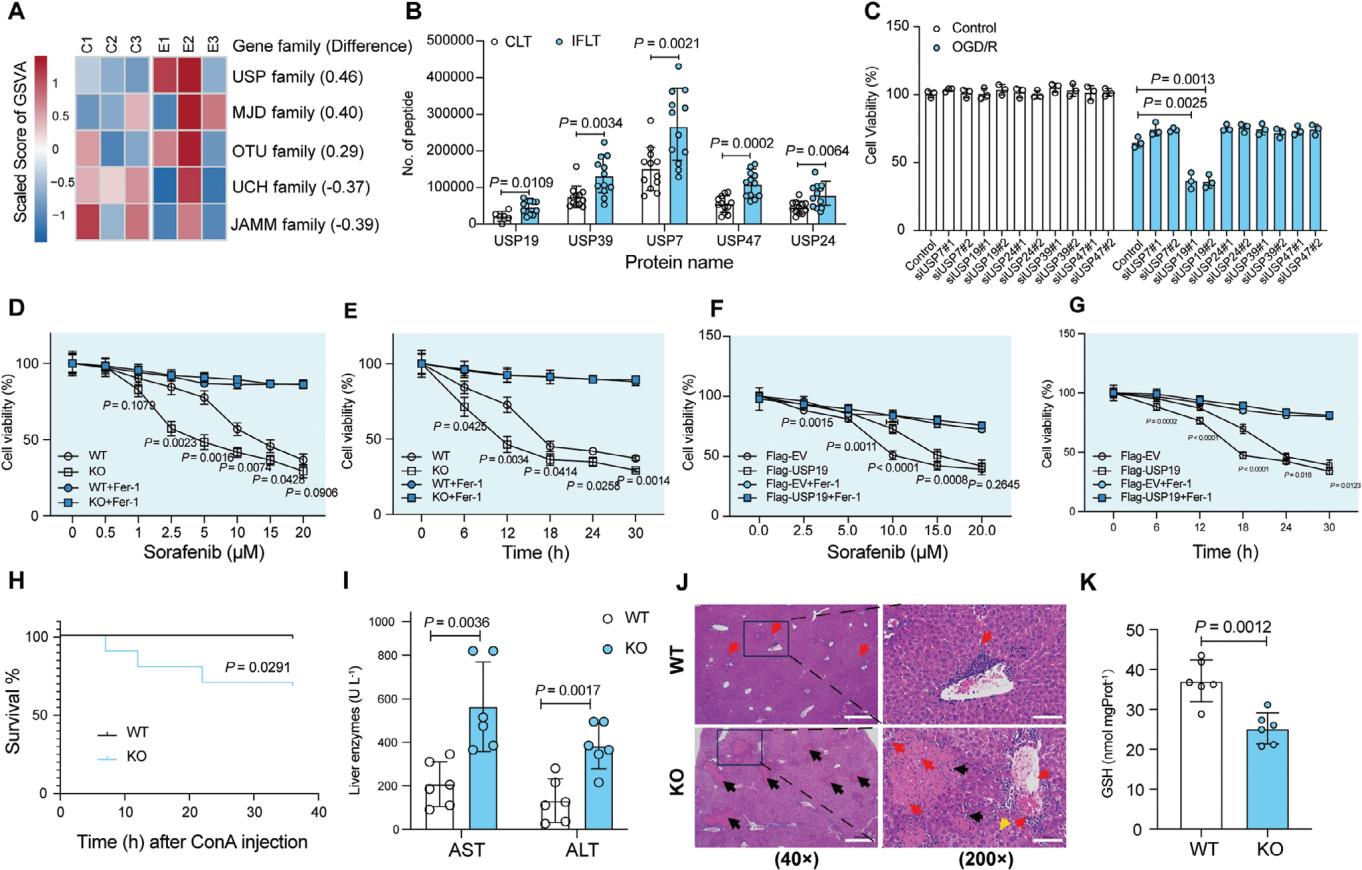
Deubiquitinase USP19 suppresses ferroptosis of hepatocytes. A) The GSVA scores for each deubiquitinase family were analyzed in Erastin‐induced hepatocyte ferroptosis. B) Proteomic analysis showed the numbers of peptide segments of 5 deubiquitinases (USP7, USP19, USP24, USP39, and USP47) were significantly higher in IFLT than in CLT grafts. C) The comparison of cell viability among MIHA cells exposed to OGD/R with knockdown of USP7, USP19, USP24, USP39, and USP47. D,E) Cell survival assay of hepatocytes treated with Sorafenib and Fer‐1 was measured using CCK‐8. Primary hepatocytes of WT or *Usp19*‐KO mice were stimulated with Sorafenib at the indicated concentrations for 12 h (D) or 2.5 µMSorafenib for different time points (E). F,G) Cell survival assay in control or *Usp19*‐overexpressed MIHA cells under Sorafenib treatment in indicated doses for 18 h (F) or 10 µM Sorafenib for a series of time points with or without Fer‐1 (5 µM) co‐treatment (G). H) Analysis of WT or KO mice survival upon treatment with a half‐lethal dose of concanavalin A (ConA) (*n* = 10 mice in each group). I) Serum levels of AST and ALT were assessed in WT or KO mice treated with ConA (*n* = 6 mice in each group). J) H&E staining of liver sections from ConA‐treated mice. Red arrows, infiltrating inflammatory cells; black arrows, cell coagulation necrosis, nuclear pyknosis, and deep staining; green arrows, swelling of liver cells with loose cytoplasm and light staining. left panel: scale bars, 500 µm, × 40 magnification; right panel: scale bars, 100 nm, × 200 magnification. (K), Total GSH levels in livers of indicated groups. The error bars represent the standard deviation of the indicated dataset. P values in (B‐C), (J), and (K) were analyzed by unpaired two‐tailed Student's t‐test (Prism; GraphPad). Two or more groups were compared by ANOVA (D‐G). The survival analysis was described by Kaplan‐Meier Curve (Prism; GraphPad) (H).

Growing evidence has demonstrated that ubiquitination and deubiquitination are involved in the regulation of ferroptosis.^[^ ^25^, ^26^ ^]^ We thus calculated the GSVA scores for each deubiquitinase family of hepatocyte ferroptosis induced by Erastin using an online database (GSE104462) and found that the USP family showed the most obvious change during induction of ferroptosis among the seven deubiquitinase families (JAMM, USP, MJD, OTU, UCH, ZUP1, and MINDY) (**Figure** **3**A). Proteomic analysis identified that 5 deubiquitinases (USP7, USP19, USP24, USP39, and USP47) among 56 members in the USP family, with numbers of peptide segments significantly elevated in IFLT versus CLT grafts (Figure 3B). Further screening with siRNA demonstrated that knockdown of USP19 augmented the susceptibility of MIHA cells to OGD/R, while knockdown of the other four deubiquitinases showed a slightly higher resistance against OGD/R (Figure 3C).

Next, we tried to confirm the role of USP19 in the regulation of ferroptosis. *Usp19*‐knockout (KO) primary hepatocytes were more sensitive to ferroptosis induced by Sorafenib^[^ ^27^, ^28^ ^]^ (Figure 3D,E), while hepatocytes with *Usp19* overexpression were more resistant to ferroptosis (Figure 3F,G). Concanavalin A (ConA) is known to induce ferroptosis of hepatocytes in mice through various mechanisms including downregulation of SLC7A11 and GSH levels. Administering ConA through the tail vein of mice results in liver damage or even mortality.^[^ ^29^ ^]^ The survival analysis showed that USP19 protects mice from mortality induced by ConA (*P* = 0.0291) (Figure 3H). Serum levels of ALT (383.33 ± 104.96 U L^−1^ vs 131.67 ± 100.18 U L^−1^, *P *= 0.0017) and AST (563.33 ± 206.41 U L^−1^ vs 207.50 ± 103.19 U L^−1^, *P* = 0.0036) were significantly elevated in *Usp19*‐KO mice compared to wild type (WT) mice (Figure 3I). Consistently, macro‐ and microscopic analyses showed that the necrotic tissue area was larger in livers of *Usp19*‐KO versus WT mice when exposed to ConA (Figure 3J; Figure S3, Supporting Information). Moreover, a significant decrease of GSH was documented in *Usp19*‐KO versus WT mice (25.22 ± 3.92 µmol mgProt^−1^ vs 37.16 ± 5.20 µmol mgProt^−1^, *P* = 0.0012) (Figure 3K).

### 2.3 USP19 Expression is Associated with Ferroptosis and IRI Severity in Clinical Liver Transplantation

**Figure 4:**
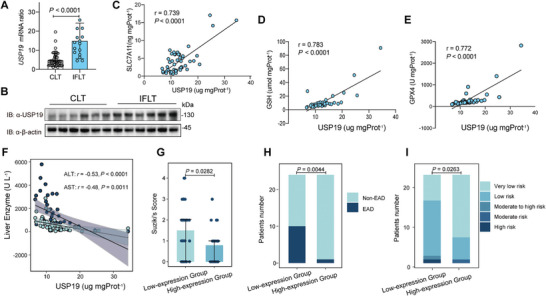
USP19 expression is upregulated in IFLT and associated with ferroptosis and clinical IRI severity. A) The changes in USP19 mRNA expression between pre‐procurement and post‐reperfusion in IFLT (*n* = 16) and CLT (*n* = 48) cohorts. USP19 mRNA ratio was calculated by the USP19 mRNA expression post‐revascularization/at preprocurement. B) The protein levels of USP19 post‐revascularization were measured in IFLT and CLT recipients (*n* = 6 in each group). C–E) Correlation analyses of USP19 protein levels against the levels of SLC7A11 (C), GSH (D), and GPX4 (E) in CLT grafts (*n* = 48). These proteins were assessed by ELISA and normalized by the total amount of proteins. F) The correlation analysis between USP19 expression and the peak serum AST and ALT within 7 days after CLT (*n* = 48). G,H) Based on the median expression of USP19 in the grafts, recipients were divided into high‐expression and low‐expression groups. Suzuki's score (G) and EAD incidence of the two groups were compared. I) The distribution of L‐GrAFT risk groups was compared between the USP19 high‐ and low‐expression groups. P values in (A) were analyzed by unpaired two‐tailed Student's t‐test (Prism; GraphPad), The comparison in the indicated groups (H) was calculated by the Wilcoxon Rank Sum Test. P values in (H) and (I) were analyzed by chi‐square test. The correlation of USP19 and SLC7A11‐GSH‐GPX4 in (C‐E), and liver enzymes in (F) were analyzed by linear regression algorithm.

To explore the role of USP19 in the regulation of ferroptosis and IRI in clinical liver transplantation, we first compared the expression of USP19 in IFLT and CLT donor livers. The mRNA level of USP19 in donor livers increased post‐reperfusion in comparison to pre‐procurement, with a more significant increase observed in the IFLT versus CLT group (**Figure** **4**A). Moreover, protein levels of USP19 post‐reperfusion also increased in the IFLT versus CLT group (Figure 4B). Importantly, the expression levels of USP19 were positively correlated with levels of anti‐ferroptosis markers, including SLC7A11 (r = 0.739, *P* < 0.0001), GSH (r = 0.783, *P* < 0.0001), and GPX4 (r = 0.772, *P* < 0.0001) in the human liver grafts (Figure 4C–E). Moreover, USP19 protein levels were negatively correlated with the peak levels of AST and ALT (r = −0.53, *P* < 0.0001 and r = −0.48, *P* < 0.0001) within the first 7 days post‐transplantation (Figure 4F). Based on the median USP19 expression (11.930 ug mgProt^−1^) of the liver samples, we divided 48 CLT recipients into high‐ and low‐expression groups. The Suzuki's scores of the donor livers, a well‐recognized criterion for the evaluation of the histological severity of IRI,^[^ ^30^ ^]^ were decreased in the USP19 high‐expression versus low‐expression groups (0.79 ± 0.80 vs 1.50 ± 1.29, *P* = 0.0282) (Figure 4G). Early allograft dysfunction (EAD) is a post‐transplant complication associated with IRI. The incidence of EAD defined by the Olthoff criteria,^[^ ^31^ ^]^ was significantly lower in the USP19 high‐expression compared to the low‐expression group (4.1% vs 41.6%, *P* = 0.0044) (Figure 4H). The L‐GrAFT risk score is a new model to assess early liver function utilizing multiple postoperative laboratory indicators to accurately predict patient outcomes.^[^ ^32^, ^33^ ^]^ The distribution of risk groups (very low risk, low risk, moderate risk, moderate‐to‐high risk, and high risk) was different between the USP19 high‐ and low‐expression groups (*P* = 0.0236). Patients with a high USP19 expression were more frequently falling in the very low‐risk group (70.8% vs 29.2%, *P* = 0.0087) (Figure 4I). Collectively, the expression of USP19 is upregulated in IFLT versus CLT grafts and closely correlated to ferroptosis and clinical IRI severity.

### 2.4 USP19 Alleviates IRI and Hepatocyte Ferroptosis in the Murine OLT Model

**Figure 5:**
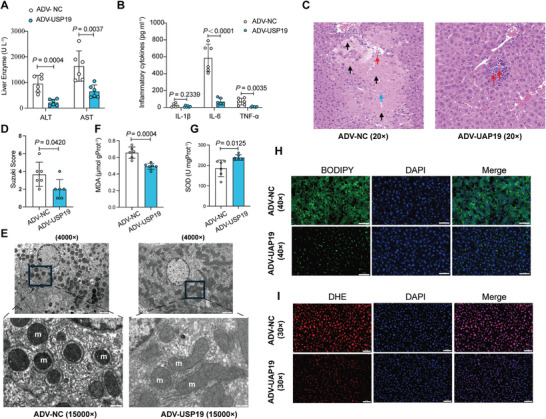
USP19 restrains IRI and inhibits ferroptosis. A) Serum AST and ALT levels in ADV‐*Usp19‐*treated and ADV‐NC‐treated mice at 6 h after OLT (*n* = 6). B) The levels of cytokines (IL‐1β, IL‐6, and TNF‐α) in ADV‐*Usp19*‐treated and ADV‐NC‐treated mice serum post‐transplantation. C,D) The representative H&E staining of liver sections (C) and the statistics of Suzuki's score for the pathological analysis (D) from ADV‐*Usp19*‐treated and ADV‐NC‐treated mice (*n* = 6). Red arrows, infiltrating inflammatory cells; black arrows, cell coagulation necrosis, nuclear pyknosis, and deep staining; blue arrows, balloon degeneration, cytoplasmic vacuolization. Scale bar, 50 µm, × 200 magnification. E) Representative hepatic TEM images of ADV‐*Usp19*‐treated group and ADV‐NC‐treated group. m, mitochondria. Top panel: scale bar, 2 µm, × 8000 magnification; bottom panel, scale bar, 500 nm, × 15 000 magnification. F,G) Levels of lipid peroxidation in livers were measured by MDA (F) and SOD (G) assay. H,I) The intracellular ROS content demonstrated by the fluorescence intensity of DHE (H) and BODIPY (I). (H), Scale bar, 50 µm, × 30 magnification. (I), Scale bar, 50 µm, × 40 magnification.

To further confirm the role of USP19‐mediated inhibition of ferroptosis in IRI, we overexpressed USP19 in the livers of mice by adenovirus (ADV) transfection and subsequently constructed murine OLT models. The serum levels of AST (657.45 ± 250.94 U L^−1^ vs 1642.05 ± 590.70 U L^−1^, *P* = 0.0023) and ALT (224.31 ± 124.61 U L^−1^ vs 957.45 ± 320.85 U L^−1^, *P* = 0.0004) at 6 h post‐transplantation were significantly decreased in ADV‐*Usp19*‐treated compared to ADV‐NC‐treated mice (**Figure** **5**A). Consistently, the levels of the serum pro‐inflammatory cytokines including IL‐6 and TNF‐α were decreased in the ADV‐*Usp19*‐treated versus ADV‐NC‐treated mice (Figure 5B). *Usp19* overexpression markedly mitigated the hepatic injury. Histological analysis showed that ADV‐NC‐treated mice exhibited an increased influx of inflammatory cells and cell necrosis compared to the overexpression group (Figure 5C). The Suzuki's score was reduced in ADV‐*Usp19*‐treated versus ADV‐NC‐treated mice (Figure 5D). Collectively, these results demonstrate the pivotal role of USP19 in reducing IRI. To further determine the difference of ferroptosis between ADV‐*Usp19*‐treated and ADV‐NC‐treated mice, we examined the mitochondrial structure. Notably, the overexpression of USP19 effectively preserved mitochondrial structure, with both the quantity of mitochondrial cristae and the density of mitochondrial membranes remaining relatively intact (Figure 5E). In contrast with the control group, the overexpression of USP19 markedly alleviated the content of MDA (0.49 ± 0.04 µmol gProt^−1^ vs 0.66 ± 0.07 µmol gProt^−1^, *P* = 0.0004) (Figure 5F) and increased that of SOD (238.65 ± 13.52 U mgProt^−1^ vs 185.36 ± 40.76 U mgProt^−1^, *P* = 0.0125) (Figure 5G) in OLT. Cytosolic ROS sensor C11‐BODIPY and DHE were used to characterize intracellular ROS. Our results indicated that ROS production was attenuated in the USP19 overexpression group compared to the control group, as manifested by weaker fluorescence intensity (Figure 5E,F). These results confirm that USP19 can suppress ferroptosis in mice OLT model.

### 2.5 USP19 Deficiency Aggravates Ferroptosis in In Vitro Cell OGD/R Injury of Primary Hepatocytes

**Figure 6:**
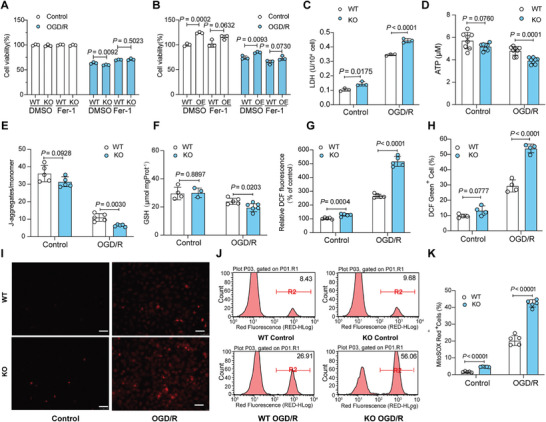
USP19 deficiency aggravates ferroptosis and cell OGD/R injury in vitro. A,B) Cell viability was evaluated by CCK8. Primary hepatocytes were isolated from WT and *Usp19*‐KO (A) or ADV‐*Usp19*‐treated and ADV‐NC‐treated (B) mice, cultured overnight and then subjected to OGD/R. Ferroptosis inhibitor (5 µm Fer‐1) was added to the culture for pretreatment prior to OGD/R. C–F) The contents of LDH (C), ATP (D), membrane potential (E) and GSH (F) were compared between the indicated groups. G–K) Cytoplasmic (G–I) and mitochondrial ROS (J,K) were detected and compared between WT and KO hepatocytes exposed to OGD/R. Cytoplasmic ROS of primary hepatocytes was detected with DCFH‐DA fluorescent probes (G,H) and DHE fluorescent probes (I). The DCF fluorescence intensity was detected by spectrophotometer (G) and flow cytometry (H) after being mounted on DCFH‐DA fluorescent probes. The intensity of red fluorescence after DHE staining also indicates ROS production (I). The bright red fluorescence was indicative of high intracellular oxidation in KO group. The primary mouse hepatocytes exposed to OGD/R were labeled with MitoSOX ™ red reagent which specifically targets mitochondria in living cells, and then the red fluorescence emitted by the reagent was detected by flow cytometry (J). The statistical analysis of the mitoSOX Red^+^ cells between groups was presented in the bar chart (K). Scale bar, 100 µm. P values were analyzed by unpaired two‐tailed Student's t‐test.

We further analyzed the regulatory roles of USP19 in ferroptosis in well‐controlled in vitro OGD/R mouse primary hepatocyte cultures. *Usp19*‐KO further exacerbated hepatocellular damage, as shown by impaired cell activity (**Figure** **6**A), higher lactate dehydrogenase (LDH) release (Figure 6C), and decreased adenosine triphosphatase (ATP) content (Figure 6D) and mitochondrial membrane potential (Figure 6E), as compared to WT group. Moreover, GSH levels were reduced in *Usp19*‐KO versus WT hepatocytes undergoing OGD/R (Figure 6F). However, overexpression of USP19 alleviated the cell damage caused by OGD/R (Figure 6B). More importantly, ferroptosis inhibitors narrowed the gap of cellular response to OGD/R when manipulating the expression of USP19 (Figure 6A,B).

Considering that endogenous ROS‐inflicted cellular damage initiates lipid peroxidation, leading to hepatocyte ferroptosis,^[^ ^34^ ^]^ the level of cytoplasmic ROS was detected using DCFH‐DA probe as quantified by spectrophotometer (Figure 5G) and flow cytometry (Figure 5H). ROS accumulation was accentuated in *Usp19*‐KO versus WT livers subsequent to OGD/R insult. Superoxide levels in hepatocytes were also detected with ROS‐sensing dye DHE. Consistently, *Usp19* deficiency significantly enhanced cellular oxidative stress (Figure 6I). Like intracellular ROS, mitochondrial ROS can also react with unsaturated fatty acids on the mitochondrial membrane, producing lipid peroxidation.^[^ ^35^ ^]^ Similarly, the mitochondria of the KO group exhibited more striking red fluorescence emitted by MitoSOX red reagent (Figure 5J,K), indicating more violent lipid peroxidation and more severe ferroptosis. Collectively, the current data show that USP19 can protect hepatocytes from OGD/R injury by inhibiting ferroptosis.

### 2.6 USP19 Inhibits Ferroptosis by Deubiquitinating SLC7A11

**Figure 7:**
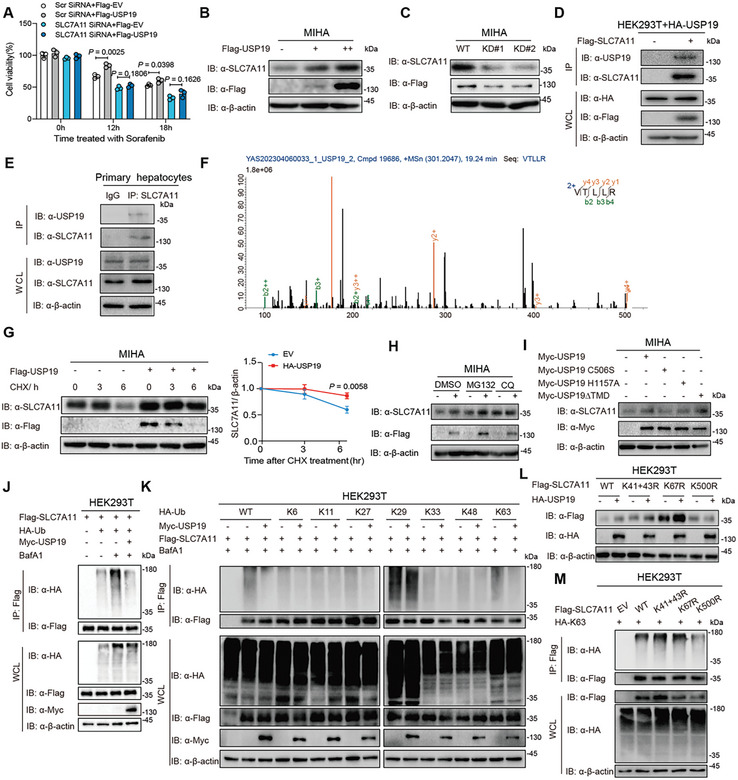
USP19 inhibits ferroptosis by deubiquitinating SLC7A11. A) The cell viability of MIHA cells treated with 10 µM Sorafenib for 0–18 h was compared between the control and USP19‐overexpression group, with or without SLC7A11 knockdown. B,C) Immunoblot analysis of SLC7A11 expression in MIHA cells with USP19 overexpression (B) or knockdown (C). D) Co‐Immunoprecipitation (Co‐IP) and immunoblot analysis of the interaction of USP19 and SLC7A11. MIHA cells were transfected with HA‐USP19 and Flag‐USP19, and then the cell extracts were harvested for analysis. E) Co‐Immunoprecipitation (Co‐IP) and immunoblot analysis of the interaction of USP19 and SLC7A11. The specific antibody against SLC7A11 (anti‐SLC7A11) was used to immunoprecipitate SLC7A11‐associated protein complexes revealing the endogenous association between SLC7A11 and USP19 in MIHA cells. F) MS analysis of proteins that interacted with USP19, including the SLC7A11 peptide. G) MIHA with or without transfected with Flag‐USP19 were treated with cycloheximide (CHX; 100 µg mL^−1^) at the indicated time points. Cell lysates were then collected for immunoblotting (left). Quantification of the protein levels of SLC7A11 by ImageJ software (NIH) (right). H) Immunoblot analysis of SLC7A11 stabilization in MIHA cells transfected with HA‐USP19, followed by DMSO, MG132 (5 µm), or CQ (50 µm) treatment for 6 h. I) Immunoblot analysis of SLC7A11 expression in MIHA cells transfected with Myc‐tagged USP19 (WT, CS, CS/HA, or ΔTMD) plasmids. J) Co‐IP and immunoblot analysis of HEK293T cells transfected with vectors for the expression of Flag‐SLC7A11 and HA‐tagged ubiquitin (Ub) in the presence of Myc‐USP19 after SDS denaturation (input: 10%). K) Lysates of HEK293T cells transfected with plasmid expressing Flag‐SLC7A11 and HA‐tagged Ub (wild type, K6‐linked‐Ub, K11‐linked‐Ub, K27‐linked‐Ub, K29‐linked‐Ub, K33‐linked‐Ub, K48‐linked‐Ub, or K63‐linked‐Ub), together with the empty vector or expression vector of Myc‐USP19 and treated with BafA1 (0.3 µM) for 6 h, were immunoprecipitated with anti‐Flag and immunoblotted with anti‐HA. L) Immunoprecipitation and immunoblot analysis of HEK293T cells transfected with vectors expressing HA‐USP19 and Flag‐SLC7A11 WT or SLC7A11 mutants. M) Co‐IP and immunoblot analysis of HEK293T cells transfected with vectors for the expression of Flag‐SLC7A11 WT, or the indicated mutants and HA‐tagged Ub in the presence of Myc‐USP19 after SDS denaturation (input: 10%). For all immunoblot data, similar results were obtained from three independent biological experiments.

Considering the positive correlation between USP19 and SLC7A11 expression in both clinical liver transplantation and mice hepatic IRI model. We thus explored whether USP19 regulates ferroptosis via SLC7A11. We found that the inhibition of Sorafenib‐induced ferroptosis by USP19 could be reversed by *Slc7a11* knockdown in MIHA cells (**Figure** **7**A). In addition, the expression of SLC7A11 was regulated by the overexpression (Figure 7B) or knockdown (Figure 7C) of USP19. To further identify the molecular mechanism by which USP19 stabilizes the SLC7A11 protein, we examined their direct interaction. Co‐immunoprecipitation (Co‐IP) and immunoblot analyses revealed that USP19 strongly interacted with SLC7A11 in HEK293T with expression vectors containing Flag‐tagged SLC7A11 together with HA‐tagged USP19 (Figure 7D). We used a specific antibody against SLC7A11 (anti‐SLC7A11) to immunoprecipitate SLC7A11‐associated protein complexes and found that the endogenous association between SLC7A11 and USP19 in MIHA cells (Figure 7E), in support of our mass spectrometry results (Figure 7F). In addition, we found that USP19 overexpression in HEK293T enhanced the stability of endogenous SLC7A11 in the presence of the translational inhibitor cycloheximide (CHX) that blocks protein synthesis, suggesting a potential inhibitory role of USP19 in SLC7A11 degradation (Figure 7G). To investigate the detailed degradation pathway of SLC7A11 regulated by USP19, we tested the protein stability of SLC7A11 with pharmacologic treatment in MIHA cells. We found that the lysosomal‐acidification inhibitor chloroquine (CQ) attenuated the influence of ectopic USP19 on SLC7A11, indicating that USP19 increases the protein level of SLC7A11 through the autophagy‐lysosome pathway (Figure 7H). To map the essential domain in which USP19 acts to stabilize SLC7A11, we generated plasmids encoding mutants of USP19 about C‐to‐S (C506S) and H‐to‐A (H1157A) substitution which are deficient in deubiquitinated activity, as well as the plasmid encoding USP19 deleted transmembrane domain (ΔTMD). As shown in Figure 7I, the catalytically inactive USP19 mutants CS and HA have been unable to stabilize SLC7A11 in contrast to USP19ΔTMD, suggesting that USP19‐mediated SLC7A11 stabilization depends on its enzyme catalytic activity of deubiquitination.

To determine whether USP19 functions as a bona fide SLC7A11 deubiquitinase, we analyzed the ubiquitination of Flag‐SLC7A11 in HEK293T by immunoprecipitation with anti‐Flag antibody, followed by immunoblot analysis with the antibody of HA‐tagged ubiquitin. These studies revealed that USP19 overexpression significantly decreased the ubiquitination of SLC7A11 (Figure 7J). Next, we delineated which type of SLC7A11 ubiquitination was removed by USP19. Overexpression of USP19 in HEK293T cells attenuated K63‐linked ubiquitination of SLC7A11, while ubiquitination of SLC7A11 with other linkages (K6, K11, K27, K29, K33, or K48) was not appreciably affected (Figure 7K). To identify the K63‐linked ubiquitination site, we constructed a series of plasmids encoding genes of SLC7A11 mutation that had indicated K‐to‐R substitutions at potential ubiquitination sites (Analyzed by http://bdmpub.biocuckoo.org/). Notably, USP19 was unable to stabilize the SLC7A11 K500R mutant in HEK293T (Figure 7L). Consistent with this observation, K63‐linked ubiquitination of the SLC7A11 K500R mutant was significantly diminished compared with WT SLC7A11 (Figure 7M). Collectively, these results suggested that USP19 protected SLC7A11 from autophagic degradation by removing K63‐linked ubiquitination at the K500 site.

### 2.7 Overexpression of USP19 Reduces IRI and Ferroptosis in a SLC7A11‐Dependent Manner

**Figure 8:**
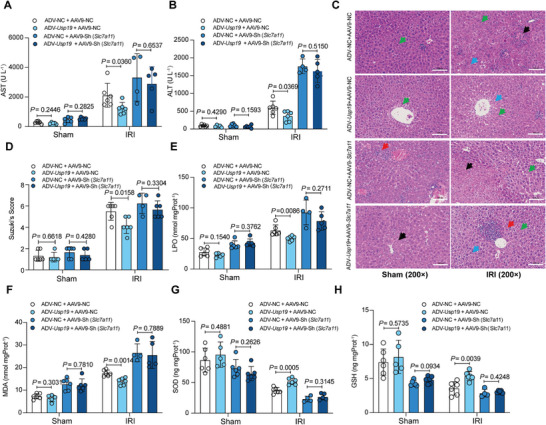
USP19 restrains IRI in a SLC7A11‐dependent manner. A–D) AAV9‐sh*Slc7a11* and ADV‐*Usp19* were injected in the lateral tail vein of mice to knockdown *Slc7a11* and overexpress *Usp19*, then mice were subjected to IRI. A,B) Serum AST (A), and ALT levels (B) were evaluated 6 h after surgery in the indicated groups. C,D) The histopathological changes, as depicted by the H&E staining images (C) and Suzuki's score (D), suggest a milder injury in the *Usp19* overexpression group. Red arrows, infiltrating inflammatory cells; black arrows, cell coagulation necrosis, nuclear pyknosis, and deep staining; green arrows, swelling of hepatocytes with loose cytoplasm and light staining; blue arrows, balloon degeneration, cytoplasmic vacuolization. Scale bar, 100 µm, × 200 magnification. E–H) The ferroptosis were evaluated by LPO (E), MDA (F), SOD (G), and GSH (H). P values in (A & B) and (E‐H) were analyzed by unpaired two‐tailed Student's t‐test (Prism; GraphPad).

Finally, we tried to test the potential clinical relevance of overexpression of USP19 in reducing hepatic IRI and inhibiting ferroptosis. We found that overexpression of USP19 by adenovirus transfection in mice significantly reduced the level of serum AST (1231.67 ± 399.37 U L^−1^ vs 2118.33 ± 803.38 U L^−1^, *P* = 0.0360) and ALT (363.33 ± 143.20 U L^−1^ vs 595.00 ± 187.38 U L^−1^, *P* = 0.0369) during partial hepatic IRI (**Figure** **8**A,B). Histopathological studies and Suzuki's scoring confirmed that USP19 overexpression can attenuate IRI (Figure 8C,D). Moreover, levels of LPO and MDA in the livers exposed to IRI were significantly reduced as a result of USP19 overexpression (Figure 8E,F). In addition, levels of SOD (51.94 ± 5.37 vs 37.13 ± 4.92 U L^−1^, *P* = 0.0005) and GSH (5.36 ± 0.67 vs 3.56 ± 0.98 U L^−1^, *P* = 0.0039) were elevated subsequent to IRI in the USP19 overexpression group in comparison to the control group (Figure 8G,H). Notably, these protections provided by the USP19 overexpression were diminished when the mice were co‐transfected with AAV‐shRNA (*Slc7a11*). Collectively, these data demonstrate that overexpression of USP19 can ameliorate IRI and inhibit ferroptosis in a SLC7A11‐dependent manner.

## 3 Discussion

IFLT has emerged as an entirely novel and promising technical innovation expanding the donor pool and improving transplant outcomes.^[^ ^11^, ^36^ ^]^ In our current study, we demonstrate that ferroptosis is substantially suppressed in IFLT versus CLT grafts. The suppression is at least partially achieved through upregulation of USP19 expression, which inhibits degradation of SLC7A11 via blocking K63‐mediated ubiquitination of the K500 site.

One of the important characteristics of graft IRI is the initiation of various programmed cell death pathways including necroptosis, apoptosis, ferroptosis, and pyroptosis.^[^ ^37^, ^38^ ^]^ Our detailed proteomic analysis in the current study showed that, among these cell death modes, ferroptosis represented the predominant form of programmed cell death after IRI in CLT grafts in comparison to IFLT grafts. Liver IRI causes ferroptosis of hepatocytes and triggers the release of cellular contents, recognized by pattern recognition receptors (PRR), including Toll‐like receptors (TLR) or Nod‐like receptors (NLR) on Kupffer cells and endothelial cells, ultimately inducing inflammatory responses and resulting in liver injury.^[^ ^39^, ^40^ ^]^ Ferroptosis inhibitor (Fer‐1) can prevent the excessive release of inflammatory cytokines during IRI, suggesting ferroptosis as an upstream event of the inflammatory response during liver IRI.^[^ ^41^ ^]^ Moreover, suppression of ferroptosis by targeting the E3 ubiquitin ligase and malic enzyme 1 (Me1) can reduce liver IRI.^[^ ^42^ ^]^ The results of the current study provide direct evidence that IFLT is able to suppress ferroptosis, thus representing one of the key mechanisms by which IFLT abrogates IRI.

Targeting specific ferroptosis core protein degradation orchestrates the complex ferroptosis response through direct or indirect regulation of iron accumulation or lipid peroxidation.^[^ ^43^ ^]^ We confirmed that the USP family was the most variable DUBs family during hepatocyte ferroptosis. Further screening found that USP19 was the only DUB member that was not only differentially expressed in IFLT and CLT donor livers, but also alleviated OGD/R injury in vitro. Moreover, our in vivo and in vitro experimental data confirm that USP19 can reduce liver IRI and alleviate ferroptosis. Of particular relevance, the expression of USP19 is negatively correlated with peak liver enzyme levels, Suzuki score, and incidence of EAD in liver transplant recipients. Taken together, our data provide evidence for the first time that USP19 can inhibit ferroptosis and reduce IRI.

Notably, even in the steady‐state condition, USP19 deficiency seems to have toxicity to the liver or hepatocyte to some extent, mainly reflecting in higher level of AST and stronger oxidative stress. In fact, the same finding has also been reported in previous literatures.^[^ ^44^, ^45^ ^]^ The injury of USP19 deficiency in the steady‐state condition is more likely due to the essential role of USP19 in maintaining cellular metabolism. USP19 is required for normal adipogenesis. When fed with a normal diet, *Usp19^−/−^* cells fail to accumulate lipids,^[^ ^45^ ^]^ which may directly influence glycogen metabolism and cellular oxidative reaction. It has also been reported that USP19 can resist the degradation of ME1 mediated by RNF1 through deubiquitination of ME1 in an unstimulated status, thereby promoting NADPH generation and maintaining ROS homeostasis,^[^ ^46^ ^]^ which is consistent with our results in Figure 5B. It is speculated that the absence of USP19 can disrupt cellular metabolic homeostasis, which is harmful to cells or mice to some extent. However, in most scenarios, this change can be compensated for.^[^ ^47^, ^48^ ^]^

The biological function of ubiquitination is largely determined by the substrates they interact with. USP19 is involved in cellular autophagy,^[^ ^49^ ^]^ inflammatory response,^[^ ^44^ ^]^ and antiviral responses^[^ ^50^ ^]^ by regulating ubiquitination modifications of BECLIN1, NLR family pyrin domain containing 3 (NLRP3), and TGF‐β activated kinase 1 (TAK1), respectively. The substrate that USP19 targets to disrupt ferroptosis is still unknown. GSH is considered as a primary antioxidant in human cells and serves as the optimal substrate for GPX4.^[^ ^51^ ^]^ The uptake of cystine, which is required for GSH formation, heavily relies on SLC7A11.^[^ ^17^ ^]^ The regulation of SLC7A11 has a direct impact on GSH and GPX4 activity, thus regulating ferroptosis.^[^ ^17^ ^]^ We have shown for the first time that USP19 interacts with and targets SLC7A11 to block K63‐mediated ubiquitination, thus preventing the degradation of SLC7A11. Furthermore, our results demonstrates that overexpression of USP19 ameliorates IRI and inhibits ferroptosis in a SLC7A11‐dependent manner. Thus, we show a novel mechanism through which USP19 suppresses ferroptosis by deubiquitinating SLC7A11.

There are limitations in the current study. First, although we are capable to establish large animal models of IFLT and CLT to validate the significant role of ferroptosis in IRI, unfortunately, at present, it is not feasible for us to manipulate gene expression in large animals, thereby making it impossible to directly verify the regulatory effect of USP19 on ferroptosis and IRI in pig models. Second, the use of an USP19 inducer in animal models might enhance the translational power of our study. However, development of USP19 inducers is still pending at this moment.

## 4 Conclusion

In conclusion, we identified ferroptosis as the most distinct programmed cell death pathway between IFLT and CLT grafts. Mechanistically, USP19 reduces ferroptosis by targeting SLC7A11 to block K63‐mediated ubiquitination, providing a new therapeutic target to reduce the detrimental consequences of liver IRI. Moreover, we have also shown an example how we can utilize IFLT as a unique clinical model to understand the molecular mechanism of graft IRI.

## 5 Experimental Section

### Patients and Samples

This study retrospectively collected data from recipients who underwent liver transplants at the Organ Transplant Center of The First Affiliated Hospital of Sun Yat‐sen University (FAH‐SYSU) from July 2021 to May 2022. All individuals who underwent CLT or IFLT during the period were eligible for inclusion in the study. The exclusion criteria were recipient age <18 years, re‐transplantation, multivisceral transplantation, split liver transplantation, living donor transplantation, and insufficient donor liver specimens retained. The liver samples used in this project were obtained at 1 h after graft revascularization.

### IFLT and CLT Models in Large White Pigs

The CLT and IFLT models in pigs were elaborated in details in the previous work.^[^ ^52^ ^]^ Six pigs were subjected to IFLT or CLT. Donor livers in IFLT underwent 8 h continuous normothermic machine perfusion (NMP) throughout graft procurement, preservation, and implantation, whereas livers in CLT were subjected to 8 h cold storage before implantation. Blood and liver specimens were obtained at 6 h post‐revascularization for further analysis.

### Murine Orthotopic Liver Transplant (OLT) Model

A well‐established mouse model was employed for ex vivo hepatic cold storage and OLT as previously described.^[^ ^53^, ^54^ ^]^ Liver grafts from male C57BL/6 mice aged 8–12 weeks were preserved in 4 °C UW solution for 18 h before being orthotopically transplanted into syngeneic male recipients aged 8–12 weeks. Liver graft and serum samples were collected 6 h post‐reperfusion, corresponding to the peak of hepatocellular damage in this model.

The model construction was divided into three steps: the procurement, back‐table preparation, and implantation of the donor livers. The detailed surgical procedures are available in the Supporting Information.

### Mouse Hepatic IRI Model

Male 6–8 weeks Usp19‐KO mice on a C57BL/6 background and wild‐type (WT) mice in the same cages were used in a partial warm hepatic IRI model. Prior to the procedure, mice were fasted but had unlimited access to water; mice were anesthetized by intraperitoneal injection of 1% pentobarbital sodium at a dose of 80 mg kg^−1^. The arterial/portal vessels to the cephalad lobes were clamped for 60 min; no vascular occlusion was performed in the sham group. Mice were anaesthetized again after 6 h of reperfusion; liver and serum samples were collected for analysis.

### Transcriptome and Proteomics Analysis

A secondary analysis of the previous proteomics and transcriptome database was conducted following the previously described analysis method.^[^ ^13^ ^]^ From the GEO database, published public transcriptome data (GSE113024 and GSE104462) was obtained. ClusterProfiler R package was used for gene name conversion. The unpaired bilateral Wilcoxon rank sum test analyzed the gene or protein expression differences. The Gene Set Variation Analysis (GSVA) was used to evaluate the enrichment of gene sets in relation to bulk RNA sequencing data. The gene dataset directly related to four programmed cell deaths was from the Integrated Biomedical Knowledgebase GeneCards Suite (https://auth.lifemapsc.com/).

### Statistical Analysis

Values were presented as the mean ± standard deviation (SD), and statistical analysis was performed using SPSS software 24.0 or GraphPad Prism 8.0. Statistical differences between the two groups were determined using an unpaired, two‐tailed student's test. For comparison among multiple groups, the analysis of variance (ANOVA) test was employed. The Chi‐Square test was used for testing the differences between categorical variables. Fisher's exact test was used when the number of variables was lower than 5. The Kaplan–Meier method was applied to generate survival curves, and the log‐rank test was used for comparison. Linear regression analysis was used to determine the correlation between the two variables.

Additional materials and methods are included in online Supporting Information.

### Ethical Statement

This study was approved and supervised by the Ethics Committee for Clinical Research and Animals Trials of FAH‐SYSU (Approval No. [2023]126). All the individuals included in this study have signed the informed consent form and agreed to participate in the research content. The animal protocol was reviewed and approved by the Institutional Animal Care and Use Committee (IACUC), SYSU (Approval NO. SYSU‐IACUC‐2022‐001962).

## Conflict of Interest

The authors declare no conflict of interest.

## Author Contributions

J.X., S.C., D.L., and Q.Z. contributed equally to this work as co‐first authors. Z.G. served as the principal investigator for the study, had full access to all of the data in the study, and took responsibility for the integrity of the data and the accuracy of the data analysis. Z.G., X.H., and J.C. conceived and supervised the project; Z.G., J.X., S.C., D.L., and Q.Z. contributed to study design, drafted the protocol, implemented the project, interpreted data, and drafted the manuscript; Y.L., H.P., and C.C., reviewed and interpreted of all pathological sections. J.X., S.C., T.L., J.Z., L.Z., and Y.C. enrolled patients, collected data, analyzed and interpreted data; Z.G., J.X., S.C., D.L., and J.C., contributed to the protocol drafting and clinical data collection; S.A., N.B., T.G.S., C.C., X.H., and Z.G. participated in paper writing and editing. All authors had access to the project results and reviewed and approved the final version of the manuscript for publication.

## Supporting information



Supporting Information

## Data Availability

The data that support the findings of this study are available from the corresponding author upon reasonable request.

## References

[1] C. Peralta , M. B. Jiménez‐Castro , J. Gracia‐Sancho , J. Hepatol. 2013, 59, 1094. 23811302 10.1016/j.jhep.2013.06.017

[2] T. Ito , B. V. Naini , D. Markovic , A. Aziz , S. Younan , M. Lu , H. Hirao , K. Kadono , H. Kojima , J. DiNorcia 3rd , V. G. Agopian , H. Yersiz , D. G. Farmer , R. W. Busuttil , J. W. Kupiec‐Weglinski , F. M. Kaldas , Am. J. Transplant. 2021, 21, 614. 32713098 10.1111/ajt.16219

[3] M. B. Jiménez‐Castro , M. E. Cornide‐Petronio , J. Gracia‐Sancho , C. Peralta , Cells 2019, 8, 1131. 31547621 10.3390/cells8101131 PMC6829519

[4] V. E. de Meijer , M. Fujiyoshi , R. J. Porte , J. Hepatol. 2019, 70, 203. 30409464 10.1016/j.jhep.2018.09.019

[5] Z. Czigany , I. Lurje , R. H. Tolba , U. P. Neumann , F. Tacke , G. Lurje , Liver Int. 2019, 39, 228. 30129192 10.1111/liv.13946

[6] P. Dutkowski , W. G. Polak , P. Muiesan , A. Schlegel , C. J. Verhoeven , I. Scalera , M. L. DeOliveira , P. Kron , P. A. Clavien , Ann. Surg. 2015, 262, 764. 26583664 10.1097/SLA.0000000000001473

[7] D. Nasralla , C. C. Coussios , H. Mergental , M. Z. Akhtar , A. J. Butler , C. D. L. Ceresa , V. Chiocchia , S. J. Dutton , J. C. García‐Valdecasas , N. Heaton , C. Imber , W. Jassem , I. Jochmans , J. Karani , S. R. Knight , P. Kocabayoglu , M. Malagò , D. Mirza , P. J. Morris , A. Pallan , A. Paul , M. Pavel , M. Perera , J. Pirenne , R. Ravikumar , L. Russell , S. Upponi , C. J. E. Watson , A. Weissenbacher , R. J. Ploeg , et al., Nature 2018, 557, 50. 29670285 10.1038/s41586-018-0047-9

[8] P. Dutkowski , J. V. Guarrera , J. de Jonge , P. N. Martins , R. J. Porte , P. A. Clavien , Gastroenterology 2019, 156, 1542. 30660724 10.1053/j.gastro.2018.12.037

[9] A. Schlegel , P. Kron , R. Graf , P. Dutkowski , P. A. Clavien , J. Hepatol. 2014, 61, 1267. 25086285 10.1016/j.jhep.2014.07.023

[10] Z. Guo , Q. Zhao , S. Huang , C. Huang , D. Wang , L. Yang , J. Zhang , M. Chen , L. Wu , Z. Zhang , Z. Zhu , L. Wang , C. Zhu , Y. Zhang , Y. Tang , C. Sun , W. Xiong , Y. Shen , X. Chen , J. Xu , T. Wang , Y. Ma , A. Hu , Y. Chen , X. Zhu , J. Rong , C. Cai , F. Gong , X. Guan , W. Huang , et al., Lancet Reg. Health West. Pac. 2021, 16, 100260. 34590063 10.1016/j.lanwpc.2021.100260 PMC8406025

[11] X. He , Z. Guo , Q. Zhao , W. Ju , D. Wang , L. Wu , L. Yang , F. Ji , Y. Tang , Z. Zhang , S. Huang , L. Wang , Z. Zhu , K. Liu , Y. Zhu , Y. Gao , W. Xiong , M. Han , B. Liao , M. Chen , Y. Ma , X. Zhu , W. Huang , C. Cai , X. Guan , X. C. Li , J. Huang , Am. J. Transplant. 2018, 18, 737. 29127685 10.1111/ajt.14583

[12] Z. Guo , Q. Zhao , Z. Jia , C. Huang , D. Wang , W. Ju , J. Zhang , L. Yang , S. Huang , M. Chen , X. Zhu , A. Hu , Y. Ma , L. Wu , Y. Chen , M. Han , Y. Tang , G. Wang , L. Wang , L. Li , W. Xiong , Z. Zhang , Y. Shen , Z. Tang , C. Zhu , X. Chen , X. Hu , Y. Guo , H. Chen , Y. Ma , et al., J. Hepatol. 2023, 79, 394.37086919

[13] Z. Guo , J. Xu , S. Huang , M. Yin , Q. Zhao , W. Ju , D. Wang , N. Gao , C. Huang , L. Yang , M. Chen , Z. Zhang , Z. Zhu , L. Wang , C. Zhu , Y. Zhang , Y. Tang , H. Chen , K. Liu , Y. Lu , Y. Ma , A. Hu , Y. Chen , X. Zhu , X. He , Clin. Transl. Med. 2022, 12, 546. 10.1002/ctm2.546 PMC9042797 35474299

[14] S. J. Dixon , K. M. Lemberg , M. R. Lamprecht , R. Skouta , E. M. Zaitsev , C. E. Gleason , D. N. Patel , A. J. Bauer , A. M. Cantley , W. S. Yang , B. Morrison 3rd , B. R. Stockwell , Cell 2012, 149, 1060. 22632970 10.1016/j.cell.2012.03.042 PMC3367386

[15] L. Galluzzi , I. Vitale , S. A. Aaronson , J. M. Abrams , D. Adam , P. Agostinis , E. S. Alnemri , L. Altucci , I. Amelio , D. W. Andrews , M. Annicchiarico‐Petruzzelli , A. V. Antonov , E. Arama , E. H. Baehrecke , N. A. Barlev , N. G. Bazan , F. Bernassola , M. J. M. Bertrand , K. Bianchi , M. V. Blagosklonny , K. Blomgren , C. Borner , P. Boya , C. Brenner , M. Campanella , E. Candi , D. Carmona‐Gutierrez , F. Cecconi , F. K. Chan , N. S. Chandel , et al., Cell Death Differ. 2018, 25, 486. 29362479 10.1038/s41418-017-0012-4 PMC5864239

[16] D. Tang , X. Chen , R. Kang , G. Kroemer , Cell Res. 2021, 31, 107. 33268902 10.1038/s41422-020-00441-1 PMC8026611

[17] Y. Ye , A. Chen , L. Li , Q. Liang , S. Wang , Q. Dong , M. Fu , Z. Lan , Y. Li , X. Liu , J. S. Ou , L. Lu , J. Yan , Kidney Int. 2022, 102, 1259. 36063875 10.1016/j.kint.2022.07.034

[18] P. Koppula , L. Zhuang , B. Gan , Protein Cell 2021, 12, 599. 33000412 10.1007/s13238-020-00789-5 PMC8310547

[19] F. He , P. Zhang , J. Liu , R. Wang , R. J. Kaufman , B. C. Yaden , M. Karin , J. Hepatol. 2023, 79, 362. 36996941 10.1016/j.jhep.2023.03.016 PMC11332364

[20] L. Song , Z. Q. Luo , J. Cell Biol. 2019, 218, 1776. 31000580 10.1083/jcb.201902074 PMC6548142

[21] L. Luo , G. Mo , D. Huang , Mol. Med. Rep. 2021, 23, 225. 33495834 10.3892/mmr.2021.11864

[22] J. Wang , Q. Zhu , Y. Wang , J. Peng , L. Shao , X. Li , Free Radic Biol. Med. 2022, 187, 171. 35660523 10.1016/j.freeradbiomed.2022.05.023

[23] C. Zhong , J. Yang , Y. Zhang , X. Fan , Y. Fan , N. Hua , D. Li , S. Jin , Y. Li , P. Chen , Y. Chen , X. Cai , Y. Zhang , L. Jiang , W. Yang , P. Yu , H. Lin , Research (Wash D C) 2023, 6, 0159. 37275121 10.34133/research.0159 PMC10232356

[24] G. Datta , B. J. Fuller , B. R. Davidson , World J. Gastroenterol. 2013, 19, 1683. 23555157 10.3748/wjg.v19.i11.1683 PMC3607745

[25] Z. Zhou , X. Song , R. Kang , D. Tang , Biomolecules 2022, 12, 1825. 36551253 10.3390/biom12121825 PMC9775562

[26] Y. Yang , M. Luo , K. Zhang , J. Zhang , T. Gao , D. O. Connell , F. Yao , C. Mu , B. Cai , Y. Shang , W. Chen , Nat. Commun. 2020, 11, 433. 31974380 10.1038/s41467-020-14324-x PMC6978386

[27] S. Yuan , C. Wei , G. Liu , L. Zhang , J. Li , L. Li , S. Cai , L. Fang , Cell Proliferation 2022, 55, 13158. 10.1111/cpr.13158 PMC8780895 34811833

[28] Q. Wang , C. Bin , Q. Xue , Q. Gao , A. Huang , K. Wang , N. Tang , Cell Death Dis. 2021, 12, 426. 33931597 10.1038/s41419-021-03718-4 PMC8087704

[29] B. Yan , Y. Ai , Q. Sun , Y. Ma , Y. Cao , J. Wang , Z. Zhang , X. Wang , Mol. Cell 2021, 81, 355. 33321093 10.1016/j.molcel.2020.11.024

[30] D. Y. Li , S. L. Xie , G. Y. Wang , X. W. Dang , Biomed. Pharmacother. 2020, 123, 109793. 31884341 10.1016/j.biopha.2019.109793

[31] K. M. Olthoff , L. Kulik , B. Samstein , M. Kaminski , M. Abecassis , J. Emond , A. Shaked , J. D. Christie , Liver Transpl. 2010, 16, 943. 20677285 10.1002/lt.22091

[32] V. G. Agopian , D. Markovic , G. B. Klintmalm , G. Saracino , W. C. Chapman , N. Vachharajani , S. S. Florman , P. Tabrizian , B. Haydel , D. Nasralla , P. J. Friend , Y. L. Boteon , R. Ploeg , M. P. Harlander‐Locke , V. Xia , J. DiNorcia , F. M. Kaldas , H. Yersiz , D. G. Farmer , R. W. Busuttil , J. Hepatol. 2021, 74, 881. 32976864 10.1016/j.jhep.2020.09.015

[33] V. G. Agopian , M. P. Harlander‐Locke , D. Markovic , W. Dumronggittigule , V. Xia , F. M. Kaldas , A. Zarrinpar , H. Yersiz , D. G. Farmer , J. R. Hiatt , R. W. Busuttil , JAMA Surg. 2018, 153, 436. 29261831 10.1001/jamasurg.2017.5040 PMC6584313

[34] K. G. Lyamzaev , A. A. Panteleeva , R. A. Simonyan , A. V. Avetisyan , B. V. Chernyak , Cells 2023, 12, 611. 36831278 10.3390/cells12040611 PMC9954536

[35] J. J. Jiang , G. F. Zhang , J. Y. Zheng , J. H. Sun , S. B. Ding , Front. Pharmacol. 2022, 13, 876550. 35496312 10.3389/fphar.2022.876550 PMC9039018

[36] M. Chen , Z. Chen , X. Lin , X. Hong , Y. Ma , C. Huang , X. He , W. Ju , Transplant Int. 2021, 34, 1261. 10.1111/tri.13828 PMC8361689 33484201

[37] M. Elias‐Miró , M. B. Jiménez‐Castro , J. Rodés , C. Peralta , Free Radic. Res. 2013, 47, 555. 23738581 10.3109/10715762.2013.811721

[38] C. Brenner , L. Galluzzi , O. Kepp , G. Kroemer , J. Hepatol. 2013, 59, 583. 23567086 10.1016/j.jhep.2013.03.033

[39] S. S. Weigt , V. Palchevskiy , J. A. Belperio , J. Clin. Invest. 2017, 127, 2022. 28569730 10.1172/JCI93537 PMC5451233

[40] J. Ding , K. Wang , W. Liu , Y. She , Q. Sun , J. Shi , H. Sun , D. C. Wang , F. Shao , Nature 2016, 535, 111. 27281216 10.1038/nature18590

[41] B. Proneth , M. Conrad , Cell Death Differ. 2019, 26, 14. 30082768 10.1038/s41418-018-0173-9 PMC6294786

[42] Y. Wu , H. Jiao , Y. Yue , K. He , Y. Jin , J. Zhang , J. Zhang , Y. Wei , H. Luo , Z. Hao , X. Zhao , Q. Xia , Q. Zhong , J. Zhang , Cell Death Differ. 2022, 29, 1705. 35260822 10.1038/s41418-022-00957-6 PMC9433446

[43] X. Chen , C. Yu , R. Kang , G. Kroemer , D. Tang , Cell Death Differ. 2021, 28, 1135. 33462411 10.1038/s41418-020-00728-1 PMC8027807

[44] T. Liu , L. Wang , P. Liang , X. Wang , Y. Liu , J. Cai , Y. She , D. Wang , Z. Wang , Z. Guo , S. Bates , X. Xia , J. Huang , J. Cui , Cell Mol. Immunol. 2021, 18, 2431. 33097834 10.1038/s41423-020-00567-7 PMC8484569

[45] E. S. Coyne , N. Bédard , Y. J. Gong , M. Faraj , A. Tchernof , S. S. Wing , Diabetologia 2019, 62, 136. 30386869 10.1007/s00125-018-4754-4

[46] Y. Zhu , L. Gu , X. Lin , X. Zhou , B. Lu , C. Liu , C. Lei , F. Zhou , Q. Zhao , E. V. Prochownik , Y. Li , Cell Rep. 2021, 37, 110174. 34965422 10.1016/j.celrep.2021.110174

[47] W. Tao , G. Zhang , C. Liu , L. Jin , X. Li , S. Yang , Redox Biol. 2023, 66, 102863. 37672892 10.1016/j.redox.2023.102863 PMC10494318

[48] M. Altun , B. Zhao , K. Velasco , H. Liu , G. Hassink , J. Paschke , T. Pereira , K. Lindsten , J. Biol. Chem. 2012, 287, 1962. 22128162 10.1074/jbc.M111.305615 PMC3265877

[49] S. Jin , S. Tian , Y. Chen , C. Zhang , W. Xie , X. Xia , J. Cui , R. F. Wang , EMBO J. 2016, 35, 866. 26988033 10.15252/embj.201593596 PMC4972138

[50] X. Zhao , Q. Di , J. Yu , J. Quan , Y. Xiao , H. Zhu , H. Li , J. Ling , W. Chen , Autophagy 2022, 18, 891. 34436957 10.1080/15548627.2021.1963155 PMC9037486

[51] F. Ursini , M. Maiorino , Free Radical Biol. Med. 2020, 152, 175. 32165281 10.1016/j.freeradbiomed.2020.02.027

[52] Y. Tang , J. Li , T. Wang , Z. Zhang , S. Huang , Z. Zhu , L. Wang , Q. Zhao , Z. Guo , X. He , Transplant Direct 2024, 10, 1597. 10.1097/TXD.0000000000001597 PMC11013694 38617464

[53] S. Kageyama , K. Nakamura , B. Ke , R. W. Busuttil , J. W. Kupiec‐Weglinski , Am. J. Transplant. 2018, 18, 1755. 29464890 10.1111/ajt.14706 PMC6035063

[54] H. Hirao , H. Kojima , K. J. Dery , K. Nakamura , K. Kadono , Y. Zhai , D. G. Farmer , F. M. Kaldas , J. W. Kupiec‐Weglinski , J. Clin. Invest. 2023, 133, 162940. 10.1172/JCI162940 PMC9888387 36719377

