# Supplementary material for: Suppression of Hepatocyte Ferroptosis via USP19‐Mediated Deubiquitination of SLC7A11 in Ischemia‐Free Liver Transplantation
Source: Adv Sci (Weinh). 2024 Nov 22;12(6):2406200. doi: 10.1002/advs.202406200 (PMC11809379; doi:10.1002/advs.202406200)
Supplement: Supplementary file 1 — Supporting Information [file ADVS-12-2406200-s001.docx]

Suppression of Hepatocyte Ferroptosis via USP19-mediated Deubiquitination of SLC7A11 in Ischemia-free Liver Transplantation

*Jinghong Xu, Shirui Chen, Di Liu, Qi Zhang, Tao Luo, Jiaxing Zhu, Liang Zhou, Yuan Lin, Hongyu Pan, Yichao Chen, Qiang Zhao, Tielong Wang, Schlegel Andrea, Björn Nashan, Tullius G. Stefan, Changjie Cai, Jun Cui, Xiaoshun He, Zhiyong Guo**

**Supplementary Information**

[Materials and Methods 3](#_Toc179973936)

[Clinical data and samples 3](#_Toc179973937)

[The definition of Ferroptosis Suppression Score 3](#_Toc179973938)

[Murine orthotopic liver transplant model 3](#_Toc179973939)

[Adenovirus/adeno-associated virus transfection 4](#_Toc179973940)

[Concanavalin A (Con A)-induced mice ferroptosis model 5](#_Toc179973941)

[Liver function assessment 5](#_Toc179973942)

[Histopathology and Suzuki’s score 5](#_Toc179973943)

[Transmission electron microscopy (TEM) 6](#_Toc179973944)

[Enzyme‐linked immunosorbent assay (ELISA) 6](#_Toc179973945)

[Lipid peroxidation assay 6](#_Toc179973946)

[Plasmids and siRNA transfection 6](#_Toc179973947)

[Mass spectrometry 7](#_Toc179973948)

[Mouse hepatocyte isolation 7](#_Toc179973949)

[OGD/R cell model 8](#_Toc179973950)

[Quantitative reverse transcription‐polymerase chain reaction (QRT-PCR) 8](#_Toc179973951)

[Co-immunoprecipitation and immunoblot analysis 8](#_Toc179973952)

[Cell viability analysis 9](#_Toc179973953)

[ROS assay 9](#_Toc179973954)

[Detection of lipid peroxides 10](#_Toc179973955)

[Supplementary Figures 10](#_Toc179973956)

[Fig. S1. Flow diagram of patient enrollment. 10](#_Toc179973957)

[Fig. S2. The levels of AST (A) and LDH (B) were compared among hepatocytes treated with Fer-1, Nec-1 and Z-VAD subjected to OGD/R. 11](#_Toc179973958)

[Fig. S3. Observation of the liver appearances of mice injected with ConA. 11](#_Toc179973959)

[Fig. S4. The vector atlas of pAAV and cADV applied in this study. 12](#_Toc179973960)

[Fig. S5. The efficacy of USP19 overexpression and SLC7A11 knockdown in mouse liver were detected. 12](#_Toc179973961)

[Supplementary Tables 13](#_Toc179973962)

[Table S1. Characteristics of donors and recipients. 13](#_Toc179973963)

[Table S2. The list of genes directly related to necroptosis, apoptosis, pyroptosis and ferroptosis 15](#_Toc179973964)

[Table S3. The siRNA sequences targeting indicated genes were listed 24](#_Toc179973965)

[Table S4. Primer sequences of certain genes used in QRT-PCR in this study were listed 26](#_Toc179973966)

# Materials and Methods

### Clinical data and samples

We compared peak levels of serum AST and ALT within 7 days, as well as TBIL on POD7 to evaluate differences in IRI of liver grafts. The occurrence of ferroptosis in the two groups was assessed by measuring GSH, MDA, and SOD content and the expression of GPX4 and SLC7A11 in corresponding donor liver samples. We detected ferroptosis and USP19 expression in 48 donor liver samples of CLT during the same period to evaluate the relationship between USP19, ferroptosis, and postoperative IRI. This study was approved and supervised by the Ethics Committee for Clinical Research and Animals Trials of FAH-SYSU (Approval NO. [2023]126).

### The definition of Ferroptosis Suppression Score

The ferroptosis suppression score is a novel biomarker identified through advancements in bioinformatics. Numerous studies have confirmed the existence of a linear relationship between ferroptosis suppression score and tumour progression, patient prognosis, and the degree of tissue damage etc.([Cong Z, et al., Apoptosis 2024](https://link.springer.com/article/10.1007/s10495-023-01900-x); [Bufu T, et al., Int J Biol Sci 2022](https://www.ncbi.nlm.nih.gov/pmc/articles/pmid/34975326/)). It was calculated by utilizing the GSVA algorithm based on the expression of ferroptosis suppressors in our clinical samples’ transcriptome dataset. The ferroptosis suppression genes were screened by FerrDb V2, which is the world’s first database (<http://www.zhounan.org/ferrdb/current/>) that dedicates to ferroptosis regulators and ferroptosis-disease associations.

### Murine orthotopic liver transplant model

Donor liver acquisition and trimming: The mouse was anaesthetised with ether inhalation, and a transverse incision was made in the upper abdomen to expose the liver. A slanted incision was created in the lower third of the common bile duct for placement and ligation of a biliary stent. 0.4 ml of normal saline containing 50 units of heparin sodium was injected into the infra-hepatic vena cava (IHVC) beneath the liver. The abdominal aorta was clamped, and 2-3 ml of 4 °C UW solution was continuously infused into the lower segment of the inferior vena cava (IVC). The superior portion of the IVC above the diaphragm was severed to allow perfusion fluid drainage. The cystic duct was ligated, and gallbladder removal followed. An incision in the IVC allowed the installation of an IVC cuff using the "double-cuff method" [2]. After uniform perfusion turned the liver light yellowish-brown, transection occurred at the portal vein (PV) above its junction with the splenic vein, followed by cuff installation on it. Below this biliary stent, disconnection occurred at the common bile duct; then suprahepatic inferior vena cava near the diaphragm cut off before retrieving the donor liver. Finally, after clamping the IHVC, it was placed in 4 °C UW solution for storage in a refrigerator.

### Adenovirus/adeno-associated virus transfection

Adenovirus (ADV) and adeno-associated virus (AAV) were employed to manipulate the expression of USP19 and SLC7A11 in mice in this study. The construction, packaging and amplification of the viral vector were accomplished by  OBiO Technology ( Shanghai ) Corp., Ltd. The interference vector pAAV TBG GdGreen WPRE was employed to construct the AAV virus. And the target sequences of NC and Slc7a11 (NM_011990.2) are GAAGTCGTGAGAAGTAGAA and TGGAGTTATACAGCTAATTAA, respectively. The plasmid of the target gene (NC: GL2004 pcADV-EF1-mScarlet-CMV-MCS-3xFLAG; Usp19: cADV-EF1-mScarlet-CMV-Usp19-3xFLAG) was recombined with the adenovirus in HEK293 cells using the AdMax system. The plasmid was validated by DNA sequencing, the Forward and Reverse primer sequence for sequencing were CGCAAATGGGCGGTAGGCGTG and GAAATTTGTGATGCTATTGC. The vector atlas of pAAV TBG GdGreen WPRE, pcADV-EF1-mScarlet-CMV-MCS-3xFLAG and cADV-EF1-mScarlet-CMV-Usp19-3xFLAG were shown in Fig. S1.

The transfection titer of ADV is 10^9, and that of AAV is 10^11. The viruses were diluted to 50 or 100 μl and injected into mice via the tail vein. Following ADV transfection into mice, the target gene commences expression within 1 - 2 days, attains its peak expression within 3 - 5 days, and can persist in expression for approximately 2 weeks; Nevertheless, when AAV is transfected into mice, the exogenous gene requires 7 - 14 days to initiate expression and reaches its peak expression after 3 - 4 weeks. Therefore, we injected AAV through the tail vein 21 days before modelling and ADV 3 days before modelling to ensure that both exogenous genes were at their peak expression during modelling.

### Concanavalin A (Con A)-induced mice ferroptosis model

Mice were weighed and given a dose of 15mg/kg Con A by injection into the tail vein. Conditions of mice within 36 hours were recorded and liver and serum samples were collected from all surviving mice by 36 hours. The Institutional Animal Care and Use Committee (IACUC), SYSU (Approval NO. SYSU-IACUC-2022-001962) reviewed and approved the animal protocol.

### Liver function assessment

The detection of serum AST, ALT, and TBIL was performed in the clinical biochemistry laboratory of FAH-SYSU with Automatic Chemical Analyzer 7600‐100 (Hitachi, Ltd, Tokyo, Japan), as described previously.

### Histopathology and Suzuki’s score

After being stored in 10% buffered formalin, liver samples were immersed in paraffin, sliced into 5 mm sections, and stained with hematoxylin and eosin (HE). Suzuki’s score was used to evaluate liver IRI calculated by two pathologists in a blinded manner. The histological changes were scored from 0 to 4 based on the degree of cytoplasmic vacuolization, sinusoidal congestion, and necrosis of parenchymal cells(1).

### Transmission electron microscopy (TEM)

To prepare ultrathin 70-nm sections for analysis using Transmission electron microscopy, liver tissues were first soaked in 2.5% glutaraldehyde in 0.1 M phosphate buffer (pH = 7.2) for over 4 hours at 4°C. They were then dehydrated in graded ethanol solutions for 10 minutes each time before being infiltrated and embedded in resin. After the sections were properly prepared and stained, images were captured through TEM (FEI Tecnai G2 Spirit Twin, Hillsboro, USA).

### Enzyme‐linked immunosorbent assay (ELISA)

The tissue was homogenized and centrifuged to obtain supernatants. Levels of GSH and inflammatory cytokines (IL-1β, IL-6, TNF-α) in liver tissues were detected using ELISA kits according to the manufacturer’s instructions (mlbio, ml063305, ml063159, ml063160, and ml002095). The absorbance of the samples was measured at 450 nm using a microplate reader, and the final concentration was calculated according to the standard curve formula.

### Lipid peroxidation assay

The tissue homogenate of concentration of SOD and MDA were assessed using Enzyme-linked immunosorbent assay kits (MIbio, ml001436; ml000439) according to the manufacturer's instructions or CuZn/Mn-SOD assay kit with WST-8 (Beyotime, S0103) and lipid peroxidation MDA assay kit (Beyotime, S0131M). And LPO was measured by a Lipid peroxidation assay kit (Nanjing Jiancheng, A106-1). Protein concentrations were determined by the BCA protein assay kit (ThermoFisher, 23227).

### Plasmids and siRNA transfection

USP19, SLC7A11, or ubiquitin constructs were cloned into the pcDNA3.1 vector and tagged with HA, Flag, or Myc. Site-directed mutagenesis was performed with the QuickChange Lightning Kit (210519-5; Agilent Technologies) according to the manufacturer’s instructions. To introduce 21-nt siRNA duplexes, Lipofectamine RNAiMAX (13778150; Invitrogen) was used for transfection, according to the manufacturer's instructions. The siRNA duplexes were chemically synthesized and obtained from OBiO (Shanghai, China). RNA oligonucleotides used are summarized in Table S3.

### Mass spectrometry

Huh7 was transfected with the plasmid of Flag-USP19 for 24 hours; then cell lysates were immunoprecipitated by Flag beads. Samples were separated by SDS–PAGE and then stained with Coomassie Blue. The entire lane was treated with trypsin at a 1:50 mass ratio to identify peptide mixtures and digested for 20 hours at 37°C. The resultant hydrolysis product was desalted, lyophilized, and stored in a 0.1% formic acid solution at -20°C. Applied Protein Technology (apt-biotech) performed liquid chromatography with tandem mass spectrometry identification. Peptides were chromatographed through an Easy-nLC 1000 system, loaded with Thermo Scientific Acclaim PepMap100, and separated by Thermo Scientific EASY column at 300 nl/min for 60 min, using a three-step acetonitrile gradient. Q Exactive mass spectrometer performed tandem MS. The RAW files generated were then analyzed by Proteome Discoverer 1.4 software for protein identification.

### Mouse hepatocyte isolation

Livers of the mice were first perfused *in situ* via the portal vein with Ca^2+^- and Mg^2+^-free Hanks’ balanced salt solution (HBSS, Gibco, 14175095) supplemented with 0.5 mM EGTA and 25 mM HEPES at 37°C. The solution was then replaced with 0.1% collagenase IV (Worthington, LS004189) in HBSS (contained Ca^2+^ and Mg^2+^, Gibco, 14025092). After a few minutes of perfusion, the liver was excised rapidly from the body cavity and placed in cold HBSS. The resulting cell suspension was filtered through a sterile 70μm pore size nylon cell strainer and centrifuged 3 times at 30×g for 4 minutes. Next, the cell precipitate was washed with percoll solution (Solarbio, P8370) to discard dead or inactive cells. The pellets were suspended in Dulbecco’s modified essential medium (DMEM, Gibco, 11965092) containing 10% fetal bovine serum (FBS, Gibco, 10099141) for primary hepatocyte culture.

### OGD/R cell model

Cell medium was removed, and hepatocytes were rinsed several times with glucose-free DMEM. To initiate oxygen–glucose deprivation, the hepatocytes were placed in a hypoxia chamber with glucose-free DMEM and an atmosphere of 5% CO_2_, 94% N_2_, and 1% O_2_ for 6h. Afterward, they were incubated at 37°C under 95% air and 5% CO_2_ for indicated hours.

### Quantitative reverse transcription‐polymerase chain reaction (QRT-PCR)

QRT-PCR was used to measure the expression intensity of different genes. TRIzol reagent (Invitrogen) was used to extract total RNA from liver tissues, and RNA extraction kits (EZBioscience, EZB-TZ1) were utilized for cells. The purity of RNA was determined using a NanoDrop 2000 spectrophotometer (Thermo Fisher Scientific, Waltham, MA, USA), and 1000 ng of RNA was reverse transcribed into complementary DNA using qPCR RT kit with gDNA remover (MIKX, MKG850-100). QRT-PCR was carried out using 2×Polarsignal® qPCR mix (MIKX, MKG800-10), following the provided instructions in a careful and precise manner. The results were analyzed using the 2^-∆∆CT^ ^(cycle threshold)^ method for quantification. RPL13A was used as an internal control. Primer sequences used in this study are summarized in Table S4.

### Co-immunoprecipitation and immunoblot analysis

Cells were lysed in low salt lysis buffer supplemented with protease inhibitor and shaken on ice for 30 min. Extracts were collected and centrifuged at 4 °C, 13,000 × g for 5 min. The supernatants were incubated with indicated antibody and Protein A/G beads overnight at 4 °C for co-IP analysis. For the ubiquitination analysis of SLC7A11, 1% SDS was added to the supernatants and boiled for 10 min. Supernatants were diluted to 0.2% SDS for immunoprecipitation. The beads were washed about 5 times and boiled with 2× SDS loading for followed SDS/PAGE. For immunoblotting analysis, 5× SDS loading was added into supernatants and boiled for 5 min for SDS/PAGE. Proteins were then transferred to PVDF membranes (Bio-Rad). After incubation with 5% skim milk and indicated antibodies, the immunoblot signal was captured by Image Lab 5.2.1 (Bio-Rad) software.

### Cell viability analysis

Cell viability was assessed using the CCK8 Cell Counting Kit (Dojindo Institute of Chemistry, CK04), following the manufacturer's guidelines. Mouse hepatocytes were cultured in a 96-well plate (Corning, 3599-1) and exposed to varying concentrations of Sorafenib for indicated duration. Subsequently, 10 μl of CCK8 reagents were added to each well, and the plate was incubated at 37°C with 5% CO_2_ for 1-4 hours then the plates were measured at 450 nm using the Tecan Safire2 Multi-Detection Microplate Reader (Morrisville, NC, USA).

### ROS assay

DHE staining

Fresh frozen liver sections were prepared and incubated with a 20 mM DHE at 37°C for 30-60 minutes. Following this, the slides were rinsed with PBS buffer, and mounted, and fluorescent images were promptly collected.

DCFH-DA or MitoSOX probes staining

The adherent cells were incubated with DCFH-DA (Nanjing Jiancheng, E004-1) or MitoSOX probes at 37°C for 20-30 minutes. After washing with PBS 3 times, the fluorescence intensity of cells was observed by a fluorescence microscope. Alternatively, the cells were collected after digestion for detection by a fluorescence spectrophotometer and flow cytometry. The fluorescence intensity was positively correlated with the level of ROS.

### Detection of lipid peroxides

Lipid peroxides were examined by C11-BODIPY 581/591 (BODIPY) staining. Briefly, liver sections were incubated with 5 µM BODIPY for 30 min at 37 ℃ in the dark. Then, the washed slides were viewed and photographed by confocal microscope to determine the intensity of green fluorescence, which was positively correlated with the level of lipid peroxides.

# [Supplementary Figures](#_Toc149679690)


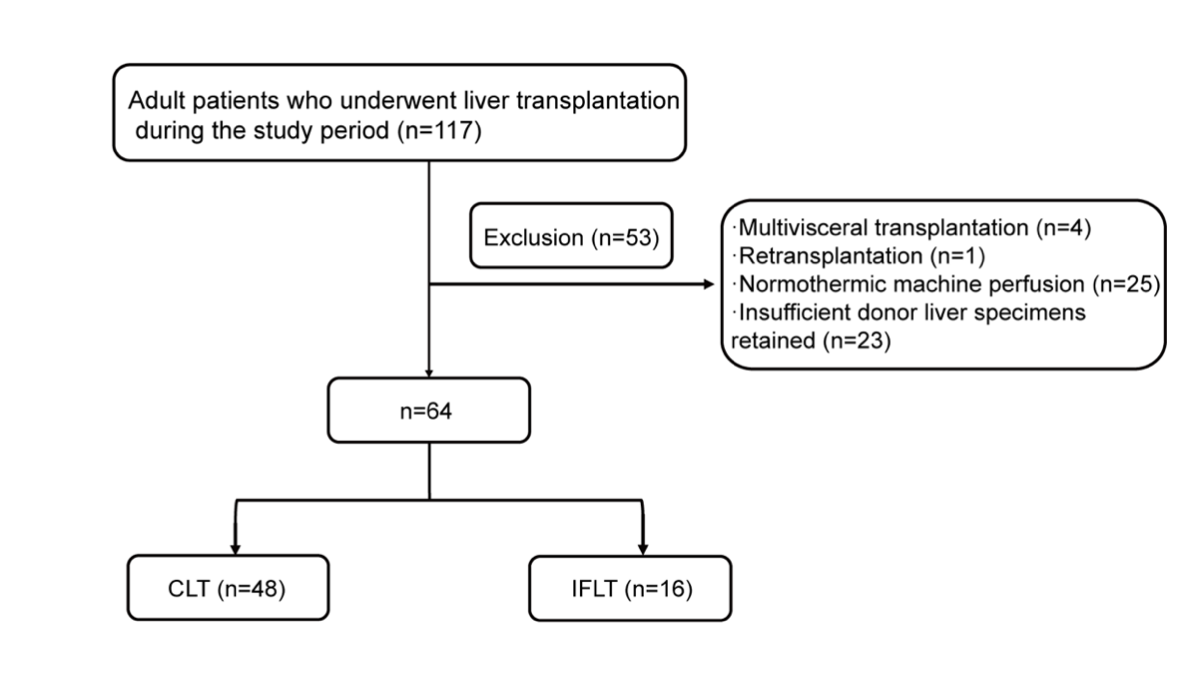


Fig. S1. Flow diagram of patient enrollment. CLT, conventional liver transplantation; IFLT, ischemia-free liver transplantation.


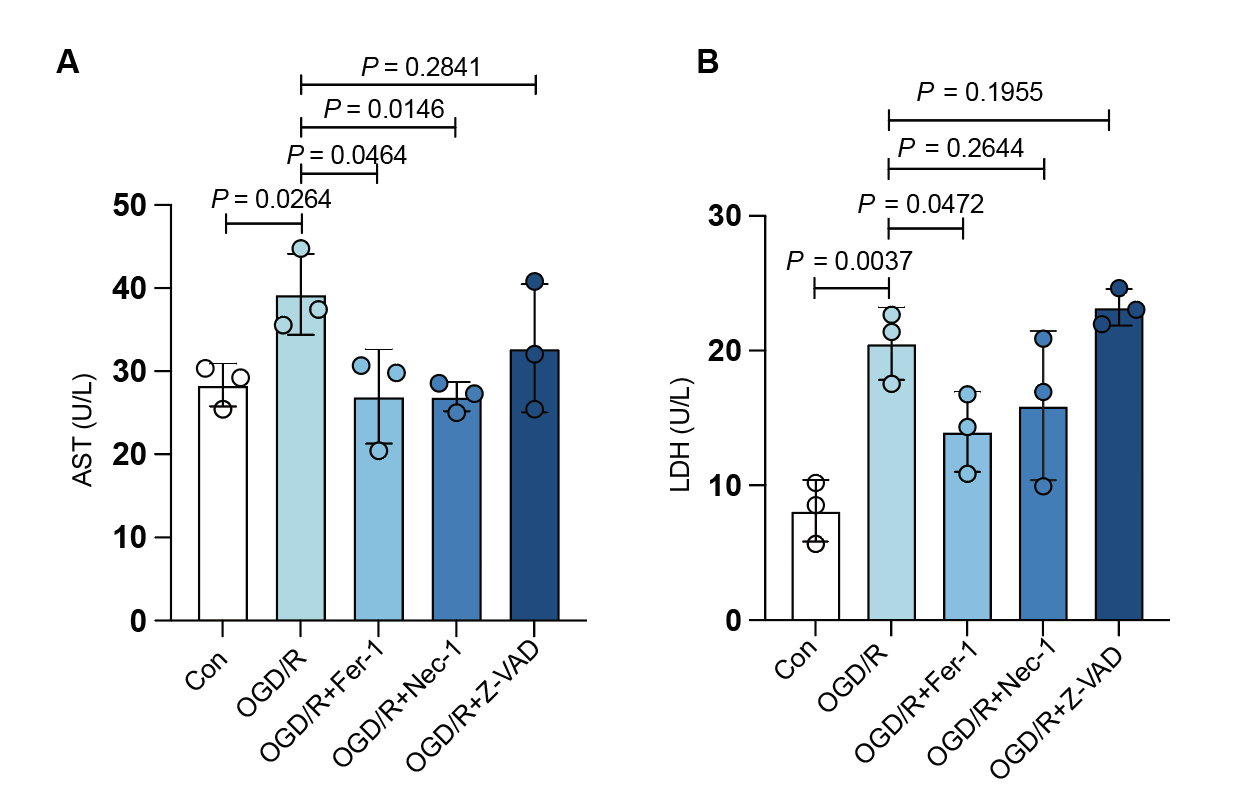


Fig. S2. The levels of AST (A) and LDH (B) were compared among hepatocytes treated with Fer-1, Nec-1 and Z-VAD subjected to OGD/R.


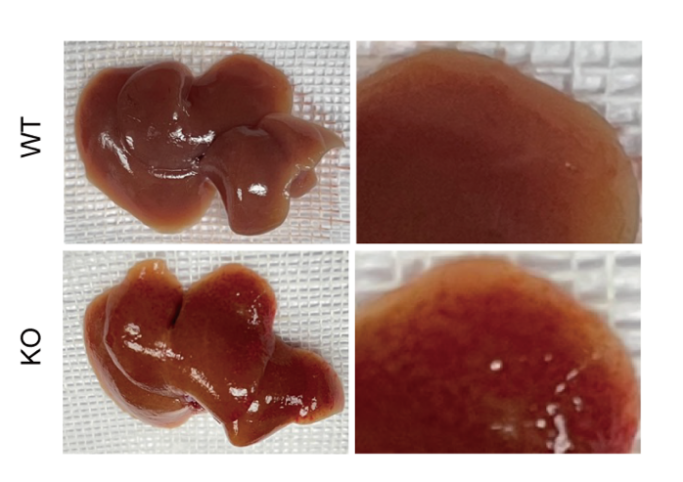


Fig. S3. Observation of the liver appearances of mice injected with ConA. The mice were sacrificed at 36 h-post injections of ConA, and liver tissues were excised for gross examination.


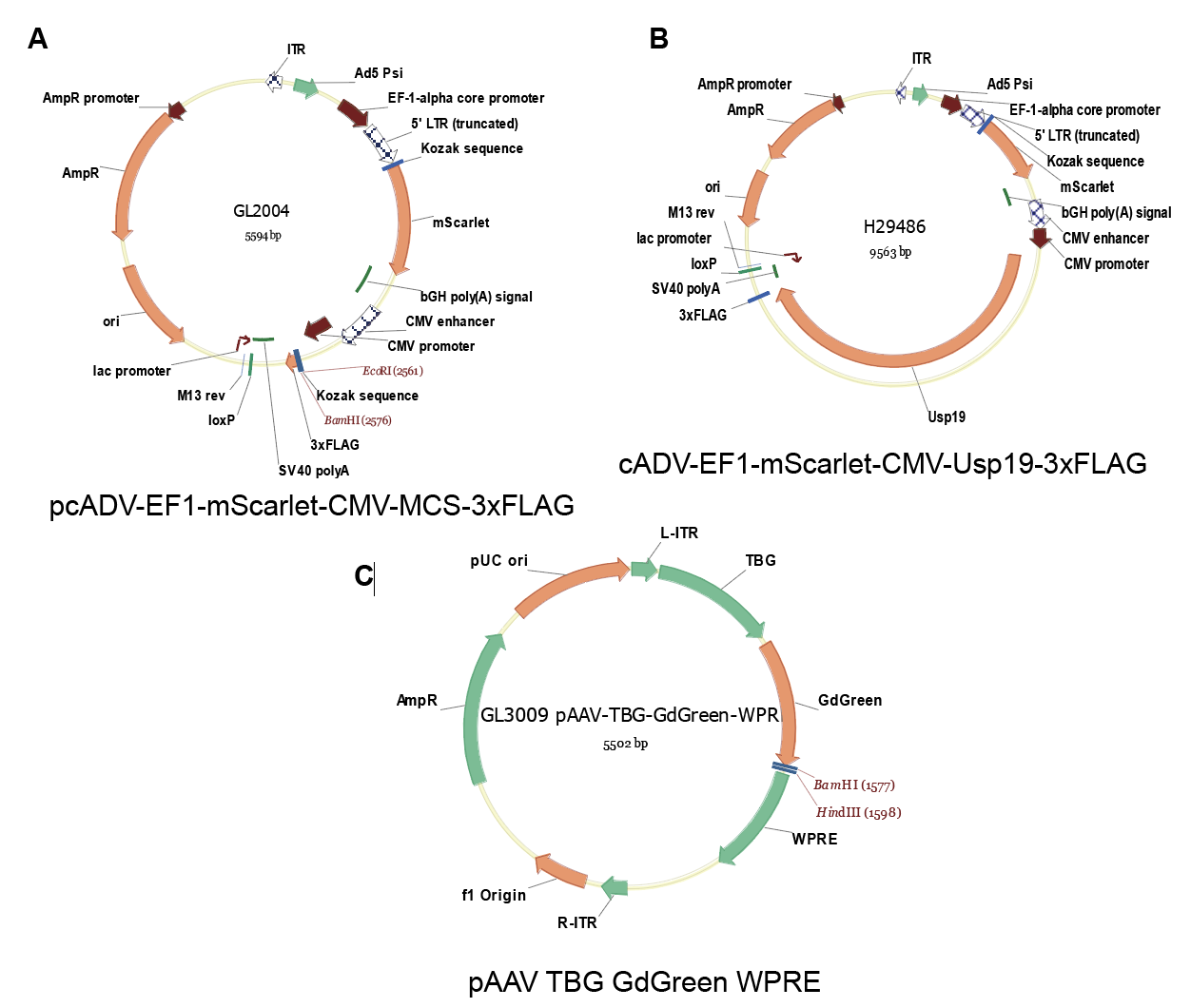


Fig. S4. The vector atlas of pAAV and cADV applied in this study.


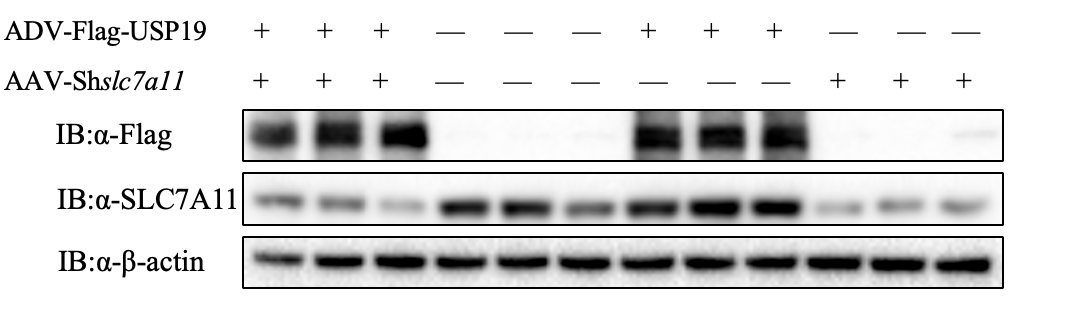


Fig. S5. The efficacy of USP19 overexpression and SLC7A11 knockdown in mouse liver were detected.

# [Supplementary Tables](#_Toc149679690)

### Table S1. Characteristics of donors and recipients.

|  | IFLT (n=16) | CLT (n=48) |
| --- | --- | --- |
| Donor characteristics |  |  |
| Age, median (IQR), yr | 46.5 (37.5-51.5) | 35 (23.5-48.3) |
| Male, No. (%) | 13 (81.3) | 42 (87.5) |
| Cause of death, No. (%) |  |  |
| CVA | 9 (56.3) | 24 (50) |
| Hypoxia | 0 (0) | 6 (12.5) |
| Trauma | 6 (37.5) | 14 (29.2) |
| Other | 1 (6.3) | 4 (8.3) |
| Body mass index, mean (SD)^a^ | 24.9 (2.6) | 22.1 (4.0) |
| Serum sodium, mean (SD) | 146.1 (8.3) | 147.8 (10.1) |
| Extended criteria donor, No. (%)^b^ | 5 (31.3) | 14 (29.2) |
| Donor risk index, mean (SD)^c^ | 1.4 (0.2) | 1.4 (0.2) |
| Cold ischemia time, median (IQR)^d^, min | NA | 338 (282-424) |
| Machine perfusion time, median (IQR)^e^, min | 229 (148-315) | NA |
| Recipient characteristics |  |  |
| Age, median (IQR), yr | 47 (41-60) | 52 (47-58) |
| Male, No. (%) | 15 (93.8) | 43 (89.6) |
| Body mass index, mean (SD) | 23.4 (3.6) | 22.9 (3.6) |
| Indication for transplantation, No. (%) |  |  |
| Hepatocellular carcinoma | 11 (68.8) | 26 (54.2) |
| Hepatitis B | 3 (18.8) | 15 (31.3) |
| Alcoholic | 1 (6.3) | 3 (6.3) |
| Miscellaneous | 1 (6.3) | 4 (8.3) |
| Laboratory MELD score, median (IQR)^f^ | 13 (7-20) | 11.5 (11-16) |
| Child-Pugh score, median (IQR)^g^ | 10 (8-12) | 9.5 (8-11) |

CLT, conventional liver transplantation; CVA, cerebrovascular accident; IFLT, ischemia-free liver transplantation; MELD, model for end-stage liver disease; NA, not applicable.

^a^The body mass index is the weight in kilograms divided by the square of the height in meters.

^b^The extended criteria donor would meet one of following criteria: Donor age >60-years-old; >25% macrovesicular steatosis; body mass index of donor >30; Before procurement, the latest serum sodium >165 mmol/L, or aspartate aminotransferase >1,000 IU/L, or alanine aminotransferase >1,000 IU/L, or total bilirubin >3 mg/dl; intensive care unit therapy over 7 days.

^c^The donor risk index is a scoring system that was developed to quantitatively predict the risk of post-transplant graft failure in liver transplantation on the basis of donor risk factors. The cold preservation time of the IFLT group is equal to zero.

^d^Cold ischemia time was defined as time between cold flush-out through aortic artery in the donor to reperfusion through portal vein in the recipient.

^e^Machine perfusion time was defined as time between machine perfusion through collateral portal vein in the donor to reperfusion through portal vein in the recipient.

^f^The MELD score of recipients is an assessment method determining organ allocation priorities for liver transplantation in the United States. The laboratory MELD score is based on original laboratory variables ranging from 6 to 40.

^g^Child-Pugh score is a clinical standard used to quantitatively evaluate liver function in cirrhotic patients, based on five indicators: hepatic encephalopathy, ascites, total bilirubin, albumin, and prothrombin time.

### Table S2. The list of genes directly related to necroptosis, apoptosis, pyroptosis and ferroptosis

| Necroptosis |  |
| --- | --- |
| [RIPK3](https://www.genecards.org/cgi-bin/carddisp.pl?gene=RIPK3&keywords=execution%20phase%20of%20necroptosis) | Receptor Interacting Serine/Threonine Kinase 3 |
| [MLKL](https://www.genecards.org/cgi-bin/carddisp.pl?gene=MLKL&keywords=execution%20phase%20of%20necroptosis) | Mixed Lineage Kinase Domain Like Pseudokinase |
| RIPK1 | Receptor Interacting Serine/Threonine Kinase 1 |
| [ZBP1](https://www.genecards.org/cgi-bin/carddisp.pl?gene=ZBP1&keywords=necroptosis) | Z-DNA Binding Protein 1 |
| [CASP8](https://www.genecards.org/cgi-bin/carddisp.pl?gene=CASP8&keywords=necroptosis) | Caspase 8 |
| [CYLD](https://www.genecards.org/cgi-bin/carddisp.pl?gene=CYLD&keywords=necroptosis) | CYLD Lysine 63 Deubiquitinase |
| [MAP3K7](https://www.genecards.org/cgi-bin/carddisp.pl?gene=MAP3K7&keywords=necroptosis) | Mitogen-Activated Protein Kinase Kinase Kinase 7 |
| [ITPK1](https://www.genecards.org/cgi-bin/carddisp.pl?gene=ITPK1&keywords=necroptosis) | Inositol-Tetrakisphosphate 1-Kinase |
| [IPMK](https://www.genecards.org/cgi-bin/carddisp.pl?gene=IPMK&keywords=necroptosis) | Inositol Polyphosphate Multikinase |
| [CASP6](https://www.genecards.org/cgi-bin/carddisp.pl?gene=CASP6&keywords=necroptosis) | Caspase 6 |
| [TRPM7](https://www.genecards.org/cgi-bin/carddisp.pl?gene=TRPM7&keywords=necroptosis) | Transient Receptor Potential Cation Channel Subfamily M Member 7 |
| [PELI1](https://www.genecards.org/cgi-bin/carddisp.pl?gene=PELI1&keywords=necroptosis) | Pellino E3 Ubiquitin Protein Ligase 1 |
| [SPATA2](https://www.genecards.org/cgi-bin/carddisp.pl?gene=SPATA2&keywords=necroptosis) | Spermatogenesis Associated 2 |
| [PGLYRP1](https://www.genecards.org/cgi-bin/carddisp.pl?gene=PGLYRP1&keywords=necroptosis) | Peptidoglycan Recognition Protein 1 |
| [FADD](https://www.genecards.org/cgi-bin/carddisp.pl?gene=FADD&keywords=necroptosis) | Fas Associated Via Death Domain |
| [HMGB1](https://www.genecards.org/cgi-bin/carddisp.pl?gene=HMGB1&keywords=necroptosis) | High Mobility Group Box 1 |
| [SIRT3](https://www.genecards.org/cgi-bin/carddisp.pl?gene=SIRT3&keywords=necroptosis) | Sirtuin 3 |
| [STING1](https://www.genecards.org/cgi-bin/carddisp.pl?gene=STING1&keywords=necroptosis) | Stimulator Of Interferon Response CGAMP Interactor 1 |
| [TNIP1](https://www.genecards.org/cgi-bin/carddisp.pl?gene=TNIP1&keywords=necroptosis) | TNFAIP3 Interacting Protein 1 |
| [MEFV](https://www.genecards.org/cgi-bin/carddisp.pl?gene=MEFV&keywords=necroptosis) | MEFV Innate Immunity Regulator, Pyrin |
| [AIM2](https://www.genecards.org/cgi-bin/carddisp.pl?gene=AIM2&keywords=necroptosis) | Absent In Melanoma 2 |
| [TNFRSF1A](https://www.genecards.org/cgi-bin/carddisp.pl?gene=TNFRSF1A&keywords=necroptosis) | TNF Receptor Superfamily Member 1A |
| [TP53](https://www.genecards.org/cgi-bin/carddisp.pl?gene=TP53&keywords=necroptosis) | Tumor Protein P53 |
| [BIRC2](https://www.genecards.org/cgi-bin/carddisp.pl?gene=BIRC2&keywords=necroptosis) | Baculoviral IAP Repeat Containing 2 |
| [TNFAIP3](https://www.genecards.org/cgi-bin/carddisp.pl?gene=TNFAIP3&keywords=necroptosis) | TNF Alpha Induced Protein 3 |
| [UCHL1](https://www.genecards.org/cgi-bin/carddisp.pl?gene=UCHL1&keywords=necroptosis) | Ubiquitin C-Terminal Hydrolase L1 |
| [SERTAD1](https://www.genecards.org/cgi-bin/carddisp.pl?gene=SERTAD1&keywords=necroptosis) | SERTA Domain Containing 1 |
| [NFKB1](https://www.genecards.org/cgi-bin/carddisp.pl?gene=NFKB1&keywords=necroptosis) | Nuclear Factor Kappa B Subunit 1 |
| [KLHDC10](https://www.genecards.org/cgi-bin/carddisp.pl?gene=KLHDC10&keywords=necroptosis) | Kelch Domain Containing 10 |
| [SFTPA1](https://www.genecards.org/cgi-bin/carddisp.pl?gene=SFTPA1&keywords=necroptosis) | Surfactant Protein A1 |
| [TRAF2](https://www.genecards.org/cgi-bin/carddisp.pl?gene=TRAF2&keywords=necroptosis) | TNF Receptor Associated Factor 2 |
| [CTSB](https://www.genecards.org/cgi-bin/carddisp.pl?gene=CTSB&keywords=necroptosis) | Cathepsin B |
| [MAPK14](https://www.genecards.org/cgi-bin/carddisp.pl?gene=MAPK14&keywords=necroptosis) | Mitogen-Activated Protein Kinase 14 |
| [CFLAR](https://www.genecards.org/cgi-bin/carddisp.pl?gene=CFLAR&keywords=necroptosis) | CASP8 And FADD Like Apoptosis Regulator |
| Apoptosis |  |
| BAD | Bcl2-associated agonist of cell death |
| LTA | Lymphotoxin-alpha |
| BAK1 | Bcl-2 homologous antagonist/killer |
| BAX | Apoptosis regulator BAX |
| BCL10 | B-cell lymphoma/leukemia 10 |
| BID | BH3-interacting domain death agonist |
| BCL2L11 | Bcl-2-like protein 11 |
| BIK | Bcl-2-interacting killer |
| BNIP3 | BCL2/adenovirus E1B 19 kDa protein-interacting protein 3 |
| BNIP3L | BCL2/adenovirus E1B 19 kDa protein-interacting protein 3-like |
| CD27 | CD27 antigen |
| CD70 | CD70 antigen |
| CYCS | Cytochrome c |
| DFFA | DNA fragmentation factor subunit alpha |
| DIABLO | Diablo IAP-binding mitochondrial protein |
| FASLG | Tumor necrosis factor receptor superfamily member 6 |
| GADD45A | Growth arrest and DNA damage-inducible protein GADD45 alpha |
| HRK | Activator of apoptosis harakiri |
| NOD1 | Nucleotide-binding oligomerization domain-containing protein 1 |
| PYCARD | Apoptosis-associated speck-like protein containing a CARD |
| TNFRSF9 | Tumor necrosis factor receptor superfamily member 9 |
| TNFSF10 | Tumor necrosis factor ligand superfamily member 10 |
| TNFSF8 | Tumor necrosis factor ligand superfamily member 8 |
| TP53BP2 | Apoptosis-stimulating of p53 protein 2 |
| TRAF3 | TNF receptor-associated factor 3 |
| CSRNP3 | Cysteine/serine-rich nuclear protein 3 |
| [PRUNE2](https://www.genecards.org/cgi-bin/carddisp.pl?gene=PRUNE2&keywords=apotosis) | Protein prune homolog 2 |
| [FAS](https://www.genecards.org/cgi-bin/carddisp.pl?gene=FAS&keywords=apotosis) | Fatty acid synthase |
| CASP8 | Caspase-8 |
| HDAC4 | Histone deacetylase 4 |
| CD40 | CD40 ligand |
| TNFRSF10B | Tumor necrosis factor receptor superfamily member 10B |
| TNFRSF10D | Tumor necrosis factor receptor superfamily member 10D |
| TNFRSF10C | Tumor necrosis factor receptor superfamily member 10C |
| MIR145 | Putative methyltransferase C9orf114 |
| BCL2 | Apoptosis regulator Bcl-2 |
| SERPINF1 | Pigment epithelium-derived factor |
| BIRC5 | Baculoviral IAP repeat-containing protein 5 |
| SDHC | Succinate dehydrogenase cytochrome b560 subunit, mitochondrial |
| PTGS2 | Prostaglandin G/H synthase 2 |
| NDRG1 | Protein NDRG1 |
| EGFR | Epidermal growth factor receptor |
| AKT1 | RAC-alpha serine/threonine-protein kinase |
| STAT3 | Signal transducer and activator of transcription 3 |
| TP53 | TP53-binding protein 1 |
| NFKB1 | Nuclear factor NF-kappa-B p105 subunit |
| SOD1 | Superoxide dismutase [Cu-Zn] |
| CAT | Catalase |
| DPYD | Dihydropyrimidine dehydrogenase [NADP(+)] |
| PPP3CA | Serine/threonine-protein phosphatase |
| PTEN | Phosphatidylinositol 3,4,5-trisphosphate 3-phosphatase and dual-specificity protein phosphatase PTEN |
| ADAM17 | Disintegrin and metalloproteinase domain-containing protein 17 |
| HSP90AA1 | Heat shock protein HSP 90-alpha |
| NFKBIA | NF-kappa-B inhibitor alpha |
| TUBB | Tubulin beta chain |
| TYMS | Thymidylate synthase |
| CDKN1A | Cyclin-dependent kinase inhibitor 1 |
| PLG | Plasminogen |
| ITGAM | Integrin alpha-M |
| RB1 | Retinoblastoma-associated protein |
| BCL2L1 | Bcl-2-like protein 1 |
| GNRHR | Gonadotropin-releasing hormone receptor |
| PIN1 | Peptidyl-prolyl cis-trans isomerase NIMA-interacting 1 |
| TUBG1 | Tubulin gamma-1 chain |
| TYMP | Thymidine phosphorylase |
| ANXA5 | Annexin A5 |
| CASP9 | Caspase-9 |
| ITGAL | Integrin alpha-L |
| PRTN3 | Myeloblastin |
| SP1 | Transcription factor Sp1 |
| THBS1 | Thrombospondin-1 |
| CXCL8 | Tumor necrosis factor-inducible gene 6 protein |
| CXCR3 | C-X-C chemokine receptor type 3 |
| HIPK2 | Homeodomain-interacting protein kinase 2 |
| PCNT | Pericentrin |
| E2F1 | Transcription factor E2F1 |
| NIN | Ninein |
| TREM1 | Triggering receptor expressed on myeloid cells 1 |
| CHMP5 | Charged multivesicular body protein 5 |
| PPM1H | Protein phosphatase 1H |
| Pyroptosis |  |
| IL18 | Interleukin-18 |
| IL1b | Interleukin-1 beta |
| IL6 | Interleukin-6 |
| CASP1 | Caspase-1 |
| CASP3 | Caspase-3 |
| CASP4 | Caspase-4 |
| CASP7 | Caspase-7 |
| CASP8 | Caspase-8 |
| CASP9 | Caspase-9 |
| CASP14 | Caspase-14 |
| AIM2 | Interferon-inducible protein AIM2 |
| NLRP1 | NACHT, LRR and PYD domains-containing protein 1 |
| NLRP3 | NACHT, LRR and PYD domains-containing protein 3 |
| NLRP9 | NACHT, LRR and PYD domains-containing protein 9 |
| GSDMA | Gasdermin-A |
| GSDMC | Gasdermin-C |
| GSDMD | Gasdermin-D |
| GSDME | Gasdermin-E |
| NLRP10 | NACHT, LRR and PYD domains-containing protein 10 |
| Ferroptosis |  |
| [AIFM2](https://www.genecards.org/cgi-bin/carddisp.pl?gene=AIFM2&keywords=Ferroptosis) | Apoptosis Inducing Factor Mitochondria Associated 2 |
| [GPX4](https://www.genecards.org/cgi-bin/carddisp.pl?gene=GPX4&keywords=Ferroptosis) | Glutathione Peroxidase 4 |
| [SLC7A11](https://www.genecards.org/cgi-bin/carddisp.pl?gene=SLC7A11&keywords=Ferroptosis) | Solute Carrier Family 7 Member 11 |
| [TP53](https://www.genecards.org/cgi-bin/carddisp.pl?gene=TP53&keywords=Ferroptosis) | Tumor Protein P53 |
| [NFE2L2](https://www.genecards.org/cgi-bin/carddisp.pl?gene=NFE2L2&keywords=Ferroptosis) | NFE2 Like BZIP Transcription Factor 2 |
| [ACSL4](https://www.genecards.org/cgi-bin/carddisp.pl?gene=ACSL4&keywords=Ferroptosis) | Acyl-CoA Synthetase Long Chain Family Member 4 |
| [NCOA4](https://www.genecards.org/cgi-bin/carddisp.pl?gene=NCOA4&keywords=Ferroptosis) | Nuclear Receptor Coactivator 4 |
| [TMEM164](https://www.genecards.org/cgi-bin/carddisp.pl?gene=TMEM164&keywords=Ferroptosis) | Transmembrane Protein 164 |
| [TFRC](https://www.genecards.org/cgi-bin/carddisp.pl?gene=TFRC&keywords=Ferroptosis) | Transferrin Receptor |
| [TRIM7](https://www.genecards.org/cgi-bin/carddisp.pl?gene=TRIM7&keywords=Ferroptosis) | Tripartite Motif Containing 7 |
| [HMOX1](https://www.genecards.org/cgi-bin/carddisp.pl?gene=HMOX1&keywords=Ferroptosis) | Heme Oxygenase 1 |
| [SLC40A1](https://www.genecards.org/cgi-bin/carddisp.pl?gene=SLC40A1&keywords=Ferroptosis) | Solute Carrier Family 40 Member 1 |
| [SLC3A2](https://www.genecards.org/cgi-bin/carddisp.pl?gene=SLC3A2&keywords=Ferroptosis) | Solute Carrier Family 3 Member 2 |
| [SLC1A5](https://www.genecards.org/cgi-bin/carddisp.pl?gene=SLC1A5&keywords=Ferroptosis) | Solute Carrier Family 1 Member 5 |
| [VDAC3](https://www.genecards.org/cgi-bin/carddisp.pl?gene=VDAC3&keywords=Ferroptosis) | Voltage Dependent Anion Channel 3 |
| [ALOX15](https://www.genecards.org/cgi-bin/carddisp.pl?gene=ALOX15&keywords=Ferroptosis) | Arachidonate 15-Lipoxygenase |
| [CBS](https://www.genecards.org/cgi-bin/carddisp.pl?gene=CBS&keywords=Ferroptosis) | Cystathionine Beta-Synthase |
| [IREB2](https://www.genecards.org/cgi-bin/carddisp.pl?gene=IREB2&keywords=Ferroptosis) | Iron Responsive Element Binding Protein 2 |
| [GCLC](https://www.genecards.org/cgi-bin/carddisp.pl?gene=GCLC&keywords=Ferroptosis) | Glutamate-Cysteine Ligase Catalytic Subunit |
| [AKR1C1](https://www.genecards.org/cgi-bin/carddisp.pl?gene=AKR1C1&keywords=Ferroptosis) | Aldo-Keto Reductase Family 1 Member C1 |
| [PCBP2](https://www.genecards.org/cgi-bin/carddisp.pl?gene=PCBP2&keywords=Ferroptosis) | Poly(RC) Binding Protein 2 |
| [AKR1C2](https://www.genecards.org/cgi-bin/carddisp.pl?gene=AKR1C2&keywords=Ferroptosis) | Aldo-Keto Reductase Family 1 Member C2 |
| [HSPB1](https://www.genecards.org/cgi-bin/carddisp.pl?gene=HSPB1&keywords=Ferroptosis) | Heat Shock Protein Family B (Small) Member 1 |
| [FTH1](https://www.genecards.org/cgi-bin/carddisp.pl?gene=FTH1&keywords=Ferroptosis) | Ferritin Heavy Chain 1 |
| [POR](https://www.genecards.org/cgi-bin/carddisp.pl?gene=POR&keywords=Ferroptosis) | Cytochrome P450 Oxidoreductase |
| [PCBP1](https://www.genecards.org/cgi-bin/carddisp.pl?gene=PCBP1&keywords=Ferroptosis) | Poly(RC) Binding Protein 1 |
| [ATG7](https://www.genecards.org/cgi-bin/carddisp.pl?gene=ATG7&keywords=Ferroptosis) | Autophagy Related 7 |
| [FTMT](https://www.genecards.org/cgi-bin/carddisp.pl?gene=FTMT&keywords=Ferroptosis) | Ferritin Mitochondrial |
| [GCH1](https://www.genecards.org/cgi-bin/carddisp.pl?gene=GCH1&keywords=Ferroptosis) | GTP Cyclohydrolase 1 |
| [ACSL3](https://www.genecards.org/cgi-bin/carddisp.pl?gene=ACSL3&keywords=Ferroptosis) | Acyl-CoA Synthetase Long Chain Family Member 3 |
| [MAP1LC3B](https://www.genecards.org/cgi-bin/carddisp.pl?gene=MAP1LC3B&keywords=Ferroptosis) | Microtubule Associated Protein 1 Light Chain 3 Beta |
| [SAT1](https://www.genecards.org/cgi-bin/carddisp.pl?gene=SAT1&keywords=Ferroptosis) | Spermidine/Spermine N1-Acetyltransferase 1 |
| [CP](https://www.genecards.org/cgi-bin/carddisp.pl?gene=CP&keywords=Ferroptosis) | Ceruloplasmin |
| [CISD1](https://www.genecards.org/cgi-bin/carddisp.pl?gene=CISD1&keywords=Ferroptosis) | CDGSH Iron Sulfur Domain 1 |
| [MAP1LC3C](https://www.genecards.org/cgi-bin/carddisp.pl?gene=MAP1LC3C&keywords=Ferroptosis) | Microtubule Associated Protein 1 Light Chain 3 Gamma |
| [AKR1C3](https://www.genecards.org/cgi-bin/carddisp.pl?gene=AKR1C3&keywords=Ferroptosis) | Aldo-Keto Reductase Family 1 Member C3 |
| [FTL](https://www.genecards.org/cgi-bin/carddisp.pl?gene=FTL&keywords=Ferroptosis) | Ferritin Light Chain |
| [STEAP3](https://www.genecards.org/cgi-bin/carddisp.pl?gene=STEAP3&keywords=Ferroptosis) | STEAP3 Metalloreductase |
| [NOX4](https://www.genecards.org/cgi-bin/carddisp.pl?gene=NOX4&keywords=Ferroptosis) | NADPH Oxidase 4 |
| [VDAC2](https://www.genecards.org/cgi-bin/carddisp.pl?gene=VDAC2&keywords=Ferroptosis) | Voltage Dependent Anion Channel 2 |
| [MAP1LC3A](https://www.genecards.org/cgi-bin/carddisp.pl?gene=MAP1LC3A&keywords=Ferroptosis) | Microtubule Associated Protein 1 Light Chain 3 Alpha |
| [ATG5](https://www.genecards.org/cgi-bin/carddisp.pl?gene=ATG5&keywords=Ferroptosis) | Autophagy Related 5 |
| [BECN1](https://www.genecards.org/cgi-bin/carddisp.pl?gene=BECN1&keywords=Ferroptosis) | Beclin 1 |
| [ACSL1](https://www.genecards.org/cgi-bin/carddisp.pl?gene=ACSL1&keywords=Ferroptosis) | Acyl-CoA Synthetase Long Chain Family Member 1 |
| [TF](https://www.genecards.org/cgi-bin/carddisp.pl?gene=TF&keywords=Ferroptosis) | Transferrin |
| [YAP1](https://www.genecards.org/cgi-bin/carddisp.pl?gene=YAP1&keywords=Ferroptosis) | Yes1 Associated Transcriptional Regulator |
| [PLXNB2](https://www.genecards.org/cgi-bin/carddisp.pl?gene=PLXNB2&keywords=Ferroptosis) | Plexin B2 |
| [OTUB1](https://www.genecards.org/cgi-bin/carddisp.pl?gene=OTUB1&keywords=Ferroptosis) | OTU Deubiquitinase, Ubiquitin Aldehyde Binding 1 |
| [FBXW7](https://www.genecards.org/cgi-bin/carddisp.pl?gene=FBXW7&keywords=Ferroptosis) | F-Box And WD Repeat Domain Containing 7 |
| [ATF4](https://www.genecards.org/cgi-bin/carddisp.pl?gene=ATF4&keywords=Ferroptosis) | Activating Transcription Factor 4 |
| [PRNP](https://www.genecards.org/cgi-bin/carddisp.pl?gene=PRNP&keywords=Ferroptosis) | Prion Protein |
| [KEAP1](https://www.genecards.org/cgi-bin/carddisp.pl?gene=KEAP1&keywords=Ferroptosis) | Kelch Like ECH Associated Protein 1 |
| [PARK7](https://www.genecards.org/cgi-bin/carddisp.pl?gene=PARK7&keywords=Ferroptosis) | Parkinsonism Associated Deglycase |
| [FDFT1](https://www.genecards.org/cgi-bin/carddisp.pl?gene=FDFT1&keywords=Ferroptosis) | Farnesyl-Diphosphate Farnesyltransferase 1 |
| [TXNRD1](https://www.genecards.org/cgi-bin/carddisp.pl?gene=TXNRD1&keywords=Ferroptosis) | Thioredoxin Reductase 1 |
| [LPCAT3](https://www.genecards.org/cgi-bin/carddisp.pl?gene=LPCAT3&keywords=Ferroptosis) | Lysophosphatidylcholine Acyltransferase 3 |
| [WWTR1](https://www.genecards.org/cgi-bin/carddisp.pl?gene=WWTR1&keywords=Ferroptosis) | WW Domain Containing Transcription Regulator 1 |
| [PRDX6](https://www.genecards.org/cgi-bin/carddisp.pl?gene=PRDX6&keywords=Ferroptosis) | Peroxiredoxin 6 |
| [CTH](https://www.genecards.org/cgi-bin/carddisp.pl?gene=CTH&keywords=Ferroptosis) | Cystathionine Gamma-Lyase |
| [DPP4](https://www.genecards.org/cgi-bin/carddisp.pl?gene=DPP4&keywords=Ferroptosis) | Dipeptidyl Peptidase 4 |
| [CYBB](https://www.genecards.org/cgi-bin/carddisp.pl?gene=CYBB&keywords=Ferroptosis) | Cytochrome B-245 Beta Chain |
| [SLC11A2](https://www.genecards.org/cgi-bin/carddisp.pl?gene=SLC11A2&keywords=Ferroptosis) | Solute Carrier Family 11 Member 2 |
| [GSS](https://www.genecards.org/cgi-bin/carddisp.pl?gene=GSS&keywords=Ferroptosis) | Glutathione Synthetase |
| [HMGCR](https://www.genecards.org/cgi-bin/carddisp.pl?gene=HMGCR&keywords=Ferroptosis) | 3-Hydroxy-3-Methylglutaryl-CoA Reductase |
| [PHKG2](https://www.genecards.org/cgi-bin/carddisp.pl?gene=PHKG2&keywords=Ferroptosis) | Phosphorylase Kinase Catalytic Subunit Gamma 2 |
| [SLC39A14](https://www.genecards.org/cgi-bin/carddisp.pl?gene=SLC39A14&keywords=Ferroptosis) | Solute Carrier Family 39 Member 14 |
| [SLC39A8](https://www.genecards.org/cgi-bin/carddisp.pl?gene=SLC39A8&keywords=Ferroptosis) | Solute Carrier Family 39 Member 8 |
| [ACSL5](https://www.genecards.org/cgi-bin/carddisp.pl?gene=ACSL5&keywords=Ferroptosis) | Acyl-CoA Synthetase Long Chain Family Member 5 |
| [NOX1](https://www.genecards.org/cgi-bin/carddisp.pl?gene=NOX1&keywords=Ferroptosis) | NADPH Oxidase 1 |
| [SLC38A1](https://www.genecards.org/cgi-bin/carddisp.pl?gene=SLC38A1&keywords=Ferroptosis) | Solute Carrier Family 38 Member 1 |
| [GCLM](https://www.genecards.org/cgi-bin/carddisp.pl?gene=GCLM&keywords=Ferroptosis) | Glutamate-Cysteine Ligase Modifier Subunit |
| [ACSL6](https://www.genecards.org/cgi-bin/carddisp.pl?gene=ACSL6&keywords=Ferroptosis) | Acyl-CoA Synthetase Long Chain Family Member 6 |
| [COQ2](https://www.genecards.org/cgi-bin/carddisp.pl?gene=COQ2&keywords=Ferroptosis) | Coenzyme Q2, Polyprenyltransferase |
| [CHMP5](https://www.genecards.org/cgi-bin/carddisp.pl?gene=CHMP5&keywords=Ferroptosis) | Charged Multivesicular Body Protein 5 |
| [CHMP6](https://www.genecards.org/cgi-bin/carddisp.pl?gene=CHMP6&keywords=Ferroptosis) | Charged Multivesicular Body Protein 6 |
| [SAT2](https://www.genecards.org/cgi-bin/carddisp.pl?gene=SAT2&keywords=Ferroptosis) | Spermidine/Spermine N1-Acetyltransferase Family Member 2 |
| [BACH1](https://www.genecards.org/cgi-bin/carddisp.pl?gene=BACH1&keywords=Ferroptosis) | BTB Domain And CNC Homolog 1 |
| [PRKAA1](https://www.genecards.org/cgi-bin/carddisp.pl?gene=PRKAA1&keywords=Ferroptosis) | Protein Kinase AMP-Activated Catalytic Subunit Alpha 1 |
| [MDM2](https://www.genecards.org/cgi-bin/carddisp.pl?gene=MDM2&keywords=Ferroptosis) | MDM2 Proto-Oncogene |
| [LCN2](https://www.genecards.org/cgi-bin/carddisp.pl?gene=LCN2&keywords=Ferroptosis) | Lipocalin 2 |
| [SCD](https://www.genecards.org/cgi-bin/carddisp.pl?gene=SCD&keywords=Ferroptosis) | Stearoyl-CoA Desaturase |
| [RELA](https://www.genecards.org/cgi-bin/carddisp.pl?gene=RELA&keywords=Ferroptosis) | RELA Proto-Oncogene, NF-KB Subunit |
| [GOT1](https://www.genecards.org/cgi-bin/carddisp.pl?gene=GOT1&keywords=Ferroptosis) | Glutamic-Oxaloacetic Transaminase 1 |
| [PKM](https://www.genecards.org/cgi-bin/carddisp.pl?gene=PKM&keywords=Ferroptosis) | Pyruvate Kinase M1/2 |
| [IL6](https://www.genecards.org/cgi-bin/carddisp.pl?gene=IL6&keywords=Ferroptosis) | Interleukin 6 |
| [HMGB1](https://www.genecards.org/cgi-bin/carddisp.pl?gene=HMGB1&keywords=Ferroptosis) | High Mobility Group Box 1 |
| [SIRT3](https://www.genecards.org/cgi-bin/carddisp.pl?gene=SIRT3&keywords=Ferroptosis) | Sirtuin 3 |
| [SP1](https://www.genecards.org/cgi-bin/carddisp.pl?gene=SP1&keywords=Ferroptosis) | Sp1 Transcription Factor |
| [ELAVL1](https://www.genecards.org/cgi-bin/carddisp.pl?gene=ELAVL1&keywords=Ferroptosis) | ELAV Like RNA Binding Protein 1 |
| [TNFAIP3](https://www.genecards.org/cgi-bin/carddisp.pl?gene=TNFAIP3&keywords=Ferroptosis) | TNF Alpha Induced Protein 3 |
| [FZD7](https://www.genecards.org/cgi-bin/carddisp.pl?gene=FZD7&keywords=Ferroptosis) | Frizzled Class Receptor 7 |
| [PANX1](https://www.genecards.org/cgi-bin/carddisp.pl?gene=PANX1&keywords=Ferroptosis) | Pannexin 1 |
| [ATF3](https://www.genecards.org/cgi-bin/carddisp.pl?gene=ATF3&keywords=Ferroptosis) | Activating Transcription Factor 3 |

### Table S3. The siRNA sequences targeting indicated genes were listed

|  | Oligonucleotides Sequence |
| --- | --- |
| Negative Control |  |
| Forward | 5′-UUCUCCGAACGUGUCACGUTT-3′ |
| Reverse | 5′-ACGUGACACGUUCGGAGAATT-3′ |
| Human *USP7#1* |  |
| Forward | 5′-AGUGUAAAGAAGUAGACUAUCTT-3′ |
| Reverse | 5′-GUAAGUCUAAUUAUUUACACUTT-3′ |
| Human *USP7#2* |  |
| Forward | 5′-AGAUCAUGAUGCAAUGUUAUUTT-3′ |
| Reverse | 5′-AAUAACAUUACAUCAUGAUCUTT-3′ |
| Human USP19#1 |  |
| Forward | 5′- GGAUGGAGAUCCUAGGAAAGATT-3′ |
| Reverse | 5′-UCUUUCCUAGGAUCUCCAUCCTT-3′ |
| Human USP19#2 |  |
| Forward | 5′- GGGCUGUGGAGAAGGAUAAAUTT-3′ |
| Reverse | 5′- AUUUAUCCUUCUCCACAGCCCTT-3′ |
| Human USP24#1 |  |
| Forward | 5′- AGACAACAGCAUUAUUCAAGATT-3′ |
| Reverse | 5′-UCUUGAAUAAUGCUCUUGUCUTT-3′ |
| Human USP24#2 |  |
| Forward | 5′-GGAUGUUUGUGAAUUAGAUAGTT-3′ |
| Reverse | 5′-CUAUCUAAUUCACAAACAUCCTT-3′ |
| Human USP39#1 |  |
| Forward | 5′-AGAAGACUAUUGUGACUGAUGTT-3′ |
| Reverse | 5′-CAUCAGUCACAAUAGUCUUCUTT-3′ |
| Human USP39#2 |  |
| Forward | 5′-AGAGAUUCACUAAGAACAACUTT-3′ |
| Reverse | 5′-AGUUGUUCUUAGUGAAUCUCUTT-3′ |
| Human USP47#1 |  |
| Forward | 5′-CAAUGAUGAUGGUGUUGAUGATT-3′ |
| Reverse | 5′-UCAUCACACCCAUCAUCAUUGTT-3′ |
| Human USP47#2 |  |
| Forward | 5′-AGUUCAUGUUGUUGAUCUAAATT-3′ |
| Reverse | 5′-UUUAGAUCAACAACAUGAACUTT-3′ |

### Table S4. Primer sequences of certain genes used in QRT-PCR in this study were listed

| Gene name |  |
| --- | --- |
| human *RPL13A* |  |
| Forward | 5′-GCCCTACGACAAGAAAAAGCG-3′ |
| Reverse | 5′-TACTTCCAGCCAACCTCGTGA-3′ |
| human *USP7* |  |
| Forward | 5′-GGAAGCGGGAGATACAGATGA-3′ |
| Reverse | 5′-AAGGACCGACTCACTCAGTCT-3′ |
| human *USP19* |  |
| Forward | 5′-TACCACCGCTGAACTCCCAA-3′ |
| Reverse | 5′-ACAGTCATCTTTCCCAGGGTT-3′ |
| human *USP24* |  |
| Forward | 5′-TGGACGCGGAGAAGAATGATG-3′ |
| Reverse | 5′-CTCGCTTGTAAGGGATGGACC-3′ |
| human *USP39* |  |
| Forward | 5′-GGTTTGAAGTCTCACGCCTAC-3′ |
| Reverse | 5′-GGCAGTAAAACTTGAGGGTGT-3′ |
| human *USP47* |  |
| Forward | 5′-CTCGACGCTAATTTTGAGCCA-3′ |
| Reverse | 5′-CTCTTGGAAGCGGACCTATAAAC-3′ |
| mouse *Rpl13a* |  |
| Forward | 5′-AGCCTACCAGAAAGTTTGCTTAC-3′ |
| Reverse | 5′-GCTTCTTCTTCCGATAGTGCATC-3′ |
| mouse *Ho-1* |  |
| Forward | 5′-AGGTACACATCCAAGCCGAGA-3′ |
| Reverse | 5′-CATCACCAGCTTAAAGCCTTCT-3′ |
| mouse *Ptgs2* |  |
| Forward | 5′-TGCACTATGGTTACAAAAGCTGG-3′ |
| Reverse | 5′- TCAGGAAGCTCCTTATTTCCCTT-3′ |

1. Suzuki S, Toledo-Pereyra LH, Rodriguez FJ, Cejalvo D. Neutrophil infiltration as an important factor in liver ischemia and reperfusion injury. Modulating effects of FK506 and cyclosporine. Transplantation 1993;55:1265-1272.
